# Non-Targeted Analysis Workflow of Endocrine-Disrupting Chemicals in Ovarian Follicular Fluid: Identification of Parabens by Diagnostic Fragmentation Evidence and Additional Contaminants via Mass Spectral Library Matching

**DOI:** 10.1021/acsmeasuresciau.5c00082

**Published:** 2025-08-21

**Authors:** Zilin Zhou, Michael S. Neal, Warren G. Foster, Yong-Lai Feng

**Affiliations:** 1 Exposure and Biomonitoring Division, Environmental Health Science and Research Bureau, Environmental and Radiation Health Sciences Directorate, Healthy Environments and Consumer Safety Branch, 6348 Health Canada, Ottawa, ON K1A 0K9, Canada; 2 ONE Fertility, Burlington, ON L7N 3T1, Canada; 3 Department of Obstetrics & Gynecology, 3710 McMaster University, Hamilton, ON L8S 4L8, Canada

**Keywords:** endocrine-disrupting chemicals (EDCs), non-targeted analysis (NTA), follicular fluid, parabens, phenols, high-resolution mass spectrometry (HRMS)

## Abstract

Ubiquitously distributed in the environment, food supply, and consumer products, endocrine-disrupting chemicals (EDCs) are exogenous substances that disrupt hormonal activities in the endocrine system. Increasing evidence suggests that women with reproductive disorders tend to accumulate higher levels of EDCs, such as phthalates and parabens, in ovarian follicular fluid. However, most existing studies focus on the measurements of a limited number of prevalent EDCs, overlooking chemicals and metabolites that are not known or prioritized. To address the knowledge gap, we developed a non-targeted analysis (NTA) workflow for broader EDC detection in follicular fluid samples using liquid chromatography–high-resolution mass spectrometry (LC–HRMS). By taking advantage of the higher-energy collisional dissociation (HCD) in the Orbitrap mass spectrometer, we first identified up to 17 characteristic product ions for parabens and their metabolites. Compared to conventional mass spectral matching via online databases and *in silico* fragmentation algorithms, paraben precursor ion prioritization through such diagnostic fragment ion extraction achieved more accurate compound identification at concentrations as low as 1 ng/mL. To extend the chemical coverage beyond known fragmentation patterns, we also assessed mass spectral library search via Compound Discoverer software, along with retention time model predictions. As a proof-of-concept application, the entire workflow was applied to a pooled follicular fluid sample collected from 211 Canadian patients receiving fertility treatment. Our compound identification results revealed that parabens could undergo several possible metabolic pathways, including hydrolysis, hydroxylation, sulfation, and amino acid conjugation. Furthermore, a total of 14 compounds were identified with level 1 confidence, including EDCs and their metabolites such as monophthalates, UV filters, and phenolic acids. The underlying implications of reproductive health associated with these substances are an area for future study.

## Graphical abstract

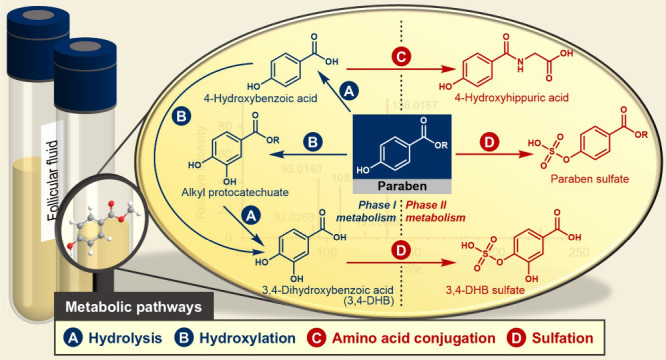

## Introduction

Identified as emerging contaminants, endocrine-disrupting chemicals (EDCs) are exogenous substances that interfere with hormone actions in the endocrine system.^1,2^ Such chemicals have been widely found in consumer products, including food, beverages, personal care products, detergents, and plasticware, entering human bodies through inhalation, ingestion, and dermal absorption.^3–5^ Human exposure to EDCs leads to increasing risks of health concerns, including neurological disorders, cardiovascular diseases, metabolic issues, obesity, and diabetes.^1,2,6^ Despite global regulatory efforts, the mass production of the synthetic EDCs results in their ubiquitous and persistent presence in the environment through airborne emissions, landfills, and wastewater release.^7–10^ The complex sources of human EDC contamination have led to significant research attention focused on analytical method development and biomonitoring in human fluid samples such as urine, blood, and saliva.^11–15^

Ovarian follicular fluid contains hormones that provide the microenvironment for the developing oocyte, whose quality is imperative for embryo development and subsequent fertility.^16^ Chemicals absorbed into bodies enter follicular fluid by passing through the blood-follicle barrier (BFB).^17^ Growing evidence has shown a direct correlation between high EDC concentrations in follicular fluid and reduced women’s fecundity.^18,19^ A case-control study by Tian et al. suggested a potentially higher risk of diminished ovarian reserve related to increased levels of 21 EDCs detected in follicular fluid.^20^ Two more recent works by Hofmann-Dishon et al.^21^ and Li et al.^17^ investigated participants’ reproductive outcomes, based on measurements of 24 and 64 EDCs in follicular fluid samples, respectively. While all these studies listed phthalates and phenolic compounds as EDC species of concern,^17,20,21^ such targeted analyses could only envelop a limited number of known chemical contaminants. Thus, a more comprehensive screening method will improve the understanding of EDC distribution in follicular fluid, especially for compounds without prior knowledge or available pure reference materials.

Non-targeted analysis (NTA) of human biological samples for exposure assessments using high-resolution mass spectrometry (HRMS) has been developing rapidly in the past decade.^22^ Typically, NTA workflows begin with considering the range of chemicals to be detected and identified (i.e., a “chemical space”)^23^ and developing a sample preparation strategy for optimal extraction. Due to rapid biotransformation processes in the human body, circulating xenobiotics such as phthalates and phenols found in urine and blood are likely metabolized.^24–30^ Specifically, using parabens as an example (Figure 1), typical metabolic processes include reduction, oxidation, or hydrolysis (phase I metabolism), as well as conjugation (phase II metabolism).^27–30^ To facilitate chemical analysis, glucuronides and sulfates are often deconjugated to their original form via sample hydrolysis, due to the unavailability of conjugate reference standards.^31,32^ After that, sample cleanup procedures such as solid-phase extraction (SPE) are often performed to eliminate interfering components in the sample matrix, notably large proteins and lipids in biological fluids.^22,33^

**Figure 1:**
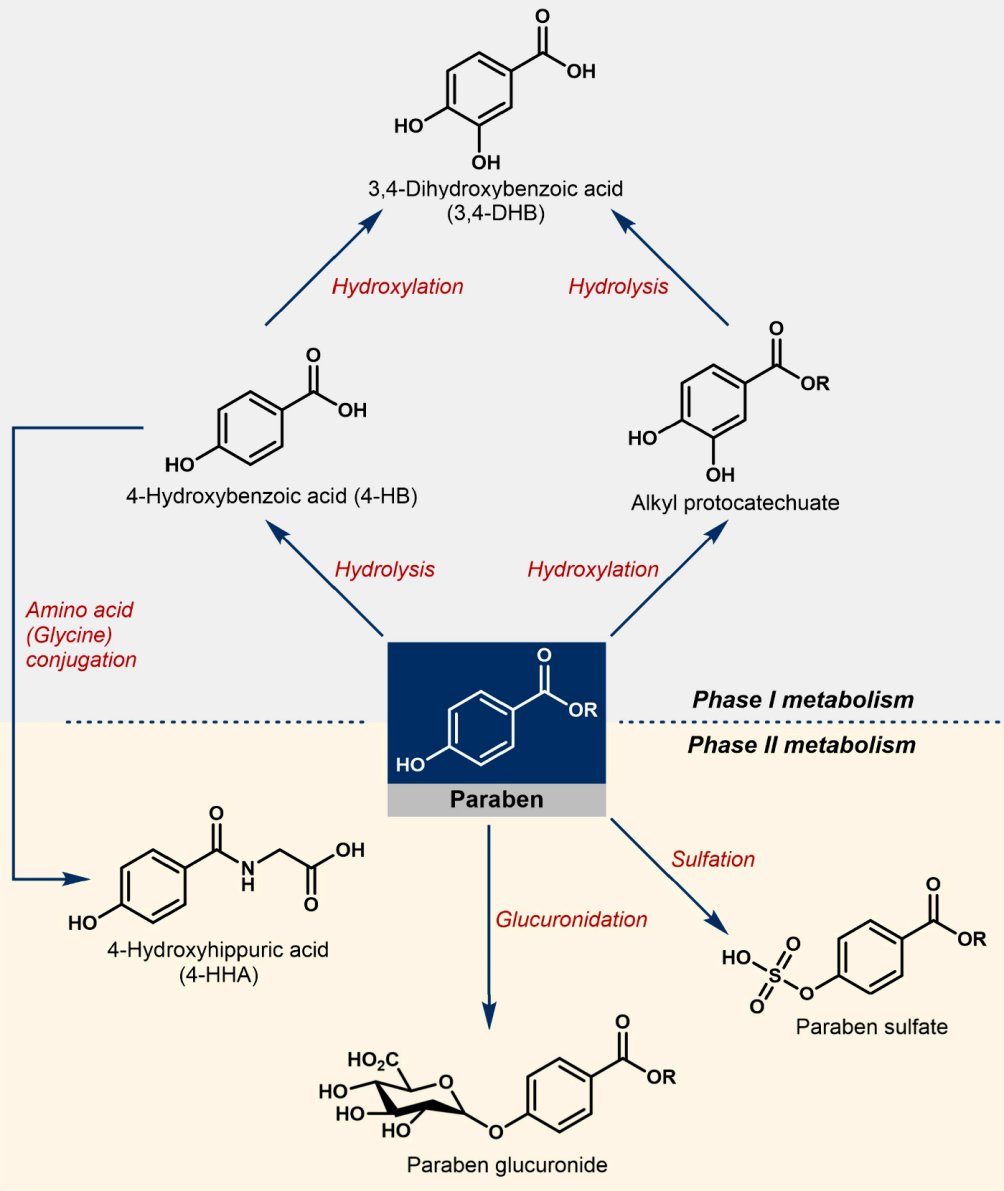
Examples of biochemical reactions occurring in the proposed metabolism of parabens. Species shown in the upper half are phase I metabolites, and the structures in the lower half are phase II metabolites.

The next stage in the NTA workflow involves liquid chromatography (LC)-HRMS data acquisition, which consists of two modes: data-dependent acquisition (DDA) and data-independent acquisition (DIA). The DDA is a product ion scan method that only fragments isolated precursor ions that meet preset requirements (typically a minimum abundance threshold) in a higher-energy collisional dissociation (HCD) cell.^22,34,35^ While this approach significantly reduces the spectral background, its constraints of a limited selection of precursor ions could overlook analytes with low concentrations, especially when multiple ions coelute. On the other hand, DIA fragments all precursor ions in the selected mass range, regardless of signal intensity.^22,34–36^ While DIA methods collect a higher volume of fragmentation data, the absence of ion selectivity can lead to high noise and lower sensitivity, along with challenges in pairing product and precursor ions in complex samples. As a result, DDA results are often supported by well-developed software, whereas unbiased DIA requires complex deconvolution methods.^37^ Nevertheless, the impacts of acquisition methods vary significantly among studies and should be examined in individual NTA method development.^37–39^

Data analysis is the final component in the NTA workflow. Here, mass spectra containing thousands of molecular features are first filtered through multiple criteria (e.g., peak intensity, mass tolerance, elemental composition, etc.) to prioritize candidate precursor ions.^22,34^ For further molecular formula assignment and structural elucidation, MS2 library searches and/or spectral matching with *in silico* fragmentation have been widely used in NTA.^22,34^ However, recent critics have highlighted the frequency of mismatches, and the reliability of identified molecular structures remains uncertain.^40–42^ Accordingly, additional confirmation tools have been established to avoid false positives, thereby enhancing the identification confidence. For example, our previous studies deployed (i) retention time (*RT*) matching via a prediction model,^43^ and (ii) manual selection of diagnostic fragment ions as constraints for monophthalate identification.^38,44^ In particular, the latter allows rapid precursor ion prioritization as all such phthalate metabolites follow similar fragmentation mechanisms.^38,44^ To expand this NTA approach of extracting chemical “fingerprints” to other types of EDCs, we hypothesize that parabens (also known as 4-hydroxybenzoates) and their metabolites can likewise be identified using diagnostic fragment ions. This is due to the fact that parabens are similar to phthalates in terms of their homologous chemical structure and phase I metabolism (see Figure 1).^27^ Therefore, we have focused on stable phenols and metabolized organic acids as our chemical space in this study, and our main objectives are to (1) characterize the fragmentation patterns for parabens and metabolites; (2) apply diagnostic ions to identify parabens and their metabolic pathways in follicular fluid; (3) compare DDA and DIA workflows for this application; and (4) evaluate the performance of NTA through computational tools for identifying a broader range of EDCs in follicular fluid.

## Materials and Methods

### Chemicals and Reagents

The detailed list of spiking chemicals, including 64 EDC reference standards and 11 isotopically labeled standards, is summarized in Tables S1 and S2, respectively. Sixteen additional standards used for retention time modeling, in-house spectral database, and/or NTA structural confirmation are listed in Table S3. Both β-glucuronidase (≥300,000 units/g) and sulfatase (≥10,000 units/g solid) enzymes were type H-1 from *Helix pomatia* (Sigma-Aldrich). Used as received, the following chemicals were HPLC or LC-MS grade: glacial acetic acid (Fisher Chemical), ammonium acetate (Sigma-Aldrich), acetonitrile (Fisher Chemical), methanol (Fisher Chemical), and water (Fisher Chemical). 800 μg/mL solutions of each standard were prepared in methanol, prior to their combination and further dilution with methanol for working solutions of native standards (20, 100, and 500 ng/mL) and a working solution of isotopically labeled standards (200 ng/mL).

### Sample collection

This study was approved by the Research Ethics Board of Health Canada (REB 2017–023H). The follicular fluid samples were collected from 211 female patients receiving reproductive treatment between 2016 and 2017 by ONE Fertility and McMaster University in Burlington, ON, Canada, for the study of the molecular physiology of human granulosa cells (HiREB 11–252-T). In this study group, the average patient age (years) was 35.7 (SD = 4.3, range = 23–45). The proportions of patients diagnosed with at least one of the following infertility conditions were: polycystic ovary syndrome (PCOS) – 6%; endometriosis – 9%; tubal factor – 11%; diminished ovarian reserve (DOR) – 29%; other female factor – 27%; male factor – 38%; unexplained – 10%. A pooled sample was prepared by combining an equal volume of follicular fluid from all of the individual patients. All samples were stored at −80 °C prior to treatment.

### Sample treatment

The sample treatment procedure was modified based on our previous work on urine^26,38,39,44^ to accommodate the removal of phospholipids and triglycerides in follicular fluid.^45,46^ A schematic diagram of the entire workflow designed for this study is shown in Figure 2. Briefly, thawed at room temperature, each 100 μL pooled follicular fluid aliquot was added with 5 μL of 200 ng/mL isotopically labeled EDC standard solution. To hydrolyze the conjugated species in follicular fluid, 100 μL of 1 M ammonium acetate solution (pH = 5.0) containing 2800 U/mL β-glucuronidase and 280 U/mL sulfatase was added. The hydrolysis was done by incubating the mixture at 37 °C for 120 min in a dry block incubator (Eppendorf Thermomixer R) at a mixing speed of 550 rpm, followed by incubation at 60 °C for 15 min to deactivate the enzymes. After cooling to room temperature, the hydrolyzed samples were acidified by an addition of 20 μL of acetic acid and 880 μL of cold acetonitrile (−20 °C) to facilitate protein precipitation. These steps were also applied to three levels of spiked samples (1, 5, and 25 ng/mL follicular fluid), which were prepared by spiking 5 μL of 20, 100, and 500 ng/mL mixed EDC standard solution respectively, to the 100 μL follicular fluid aliquots. 100 μL LC-MS grade water was used as procedural blanks. Three replicates were prepared for blanks, nonspiked samples and samples at each spiking level.

**Figure 2:**
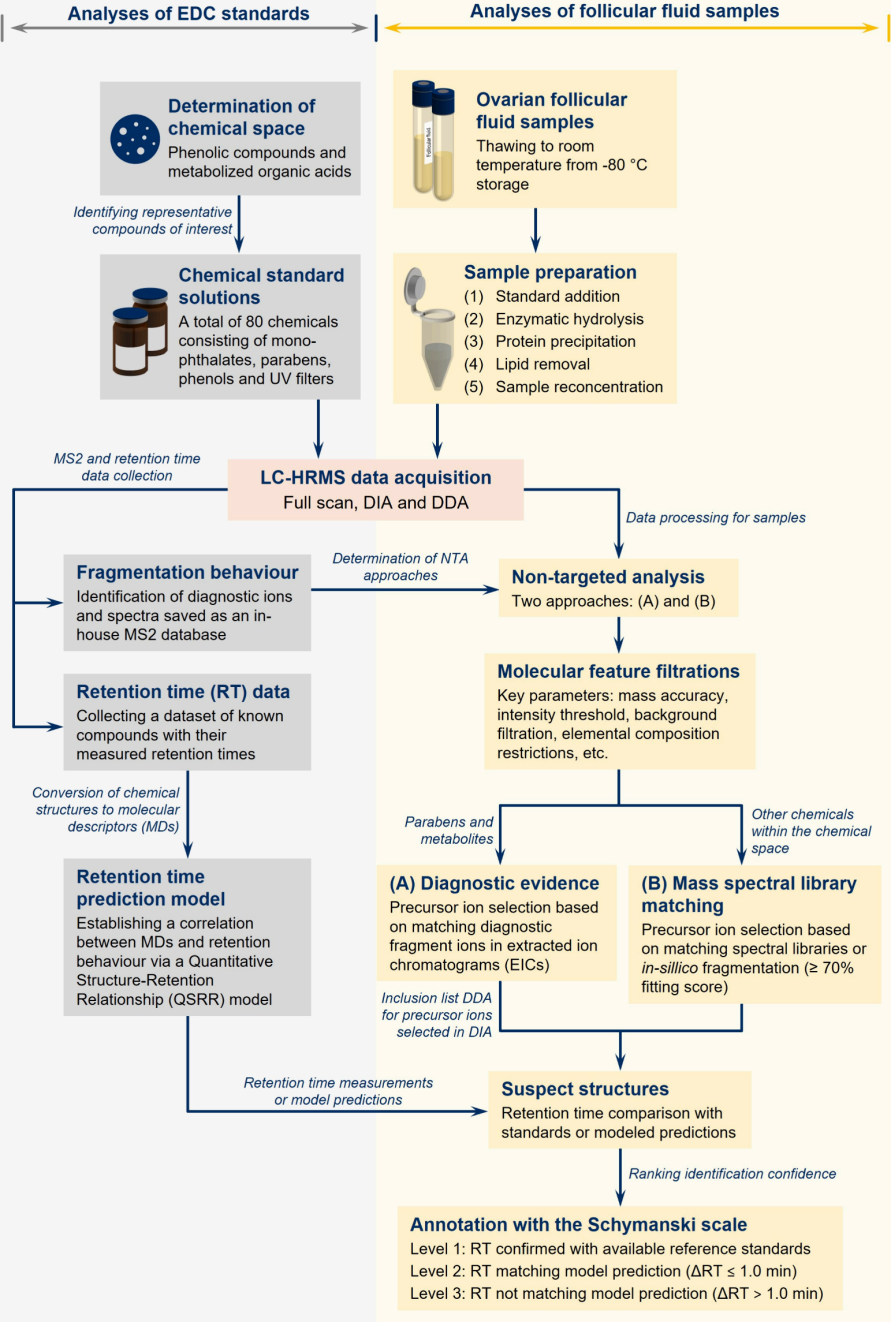
Schematic diagram of the designed workflow highlighting the key steps in this study.

After the addition of acetonitrile, each sample was further incubated in the −20 °C freezer for at least 60 min and was centrifuged at 5000 rpm for 15 min (Eppendorf centrifuge 5424). The supernatant was further filtered through a Captiva EMR-Lipid cartridge (Agilent, 40 mg/1 mL) that was preconditioned with 1 mL of 80% acetonitrile in water (0.1% acetic acid) for passthrough removal of lipids. The precipitated protein pellet was further washed with 200 μL of 80% acetonitrile in water (0.1% acetic acid), and the wash solution was also passed through the same cartridge for collection. After this cleanup process, the extracts were evaporated to dryness with a gentle flow of nitrogen gas at room temperature (Thermo Scientific Reacti-Vap evaporator). Lastly, the dried samples were reconstituted with 200 μL of 50% methanol in water (0.1% acetic acid), followed by filtration by 0.22 μM centrifugal filter (PTFE membrane) and centrifuging at 10000 rpm for 2 min (Eppendorf centrifuge 5424). To improve the spectral quality for unknown product identification, additional 500 μL nonspiked pooled follicular fluid samples were treated with and without enzymatic hydrolysis. Detailed procedures are described in Section 2 of the Supporting Information (SI).

### LC-MS data acquisition

All data acquisition was completed on a Q-Exactive Orbitrap mass spectrometer (Thermo Fisher Scientific) coupled to a Vanquish Horizon UHPLC system (Thermo Fisher Scientific). Chromatographic separation was done on an Accucore reversed phase biphenyl column (2.1 × 100 mm, 2.6 μm, Thermo Fisher Scientific) installed after an Accucore biphenyl guard cartridge (2.1 × 10 mm, 2.6 μm, Thermo Fisher Scientific). The associated mobile phases consisted of A (0.1% acetic acid in water) and B (0.1% acetic acid in methanol). With a flow rate of 0.25 mL/min, the linear solvent gradient (curve setting 5) was: 90% A and 10% B for the first minute, 40% A and 60% B at 10th minute and hold for 2 min, 15% A and 85% B at 15th minute and hold for 1 min, 10% A and 90% B at 20th minute and hold for 5 min, 90% A and 10% B at 30th minute with an additional 10 min of equilibrium time. As for the HESI ion source, monophthalates and phenolic compounds favor negative ionization, producing deprotonated molecular ions represented by [M-H]^−^.^39^ The major settings were: −2.70 kV spray voltage, 300 °C capillary temperature, 375 °C auxiliary gas heater temperature, 50.0 S-lens RF level, 40, 10, and 2 arbitrary units of sheath gas, auxiliary gas, and sweep gas flow, respectively.

MS data acquisition was done through three injections with the following scan modes: 1) full scan only, 2) DDA with full scan/ddMS2 mode, and 3) DIA with full scan/all ion fragmentation (AIF) mode. The full scan settings in all three modes were: 80 – 1000 *m*/*z* scan range, 70,000 resolution, 1e6 automatic gain control (AGC) target, and 100 ms maximum injection time. In DDA, ddMS2 settings were: 17,500 resolution, 1e4 minimum AGC target, 1e5 AGC target, 55 ms maximum injection time, 1.5 *m*/*z* isolation window, 5 loop count (TopN), and 7.0 s dynamic exclusion. In DIA, AIF settings were: 75 – 1000 *m*/*z* scan range, 35,000 resolution, 2e5 AGC target, 55 ms maximum injection time. Three scan events with 10, 20, and 40 eV collision energies (CEs) were applied in both DDA and DIA modes to maximize fragmentation ion coverage. Note that a stepped normalized CE (NCE) was not used to allow a clearer observation of fragmentation patterns at incremental CEs.

### MS/MS spectra acquisition of EDC standards

To identify the fragmentation behavior of parabens and other EDCs, a standard solution containing 50 ng/mL of 64 EDC native standards (Table S1) and 11 isotopically labeled standards (Table S2) was prepared in solvent. MS/MS spectra were acquired through the DDA mode described above with a predefined inclusion list covering precursor ion *m*/*z* values and an *RT* window of 0.4 min. For each analyte, three MS/MS spectra scanned at 10, 20, and 40 eV collision energies were saved into the mzVault software (Thermo Fisher Scientific) as in-house MS2 database for NTA.

### Data Processing: Non-Targeted Analysis of Follicular Fluid Samples

Structures of parabens and alkyl protocatechuates can be retrieved from DDA and DIA data by matching their diagnostic fragment ions in the FreeStyle Software (Thermo Fisher Scientific). Based on the investigation of their fragmentation patterns to be discussed in this study, product ions at *m*/*z* 91.0184, 95.0133, and 108.0211 were selected as the common diagnostic ions for both classes of chemicals. Additional diagnostic ions were *m*/*z* 92.0262, 93.0340, 136.0160, and 137.0239 for parabens, and *m*/*z* 109.0290, 111.0082, 124.0160, 152.0110, and 153.0188 for alkyl protocatechuates. In follicular fluid samples, prioritized precursor ions were selected when 1) peak intensity was at least 3 times higher than that of the process blanks, and 2) at least 4 diagnostic fragment ions (mass error <10 ppm) were found in the extracted ion chromatograms (EICs). Next, on Freestyle, the preselected precursor ions were further constrained through the elemental composition restrictions: 7–30 carbon, 6–60 hydrogen, 3–10 oxygen, 0–1 sulfur, 5–10 ring double bond equivalents (RDBE), and 5 ppm mass tolerance in monoisotopic mass. For DIA data, candidate precursor ions (≥10^5^ intensity at CE = 0 eV) were selected at the retention times of observed diagnostic fragment ions if their intensity consistently decreased with increasing CEs.^44^ These precursor ions were then added to an inclusion list for a separate full scan-ddMS2 acquisition to reaffirm their fragmentation for structure proposals. Lastly, if a new compound without a reference standard was proposed, retention time prediction was applied. Briefly described in Section 2 of the SI, an in-house retention time prediction model^43^ was built by converting the structures of EDC standards into molecular descriptors (inputs). A linear relationship between measured *RT*s (outputs) and a selected number of molecular descriptors was established for *RT* prediction of unknown compounds. This process is better known as a quantitative structure-retention relationship (QSRR) model.^43^

Since not all groups of endocrine-disrupting chemicals yield characteristic fragments in MS/MS, more molecular features were extracted via Compound Discoverer software 3.3.3.200 (Thermo Fisher Scientific) for a wider range of EDC identification in follicular fluid. The detailed software settings are summarized in Figure S2 and Section 2 of the SI. Here, DDA files of blanks and samples were input into the workflow. The spectral databases included the online mzCloud MS/MS library (Thermo Fisher Scientific) and the in-house mzVault library compiled in this study. For compounds without sufficient fragmentation data in spectral libraries, structural prioritization was performed through matching Human Metabolome Database (HMDB 5.0)^47^ mass lists with *in silico* fragmentation (Mass Frontier). For molecular structure annotation, a 70 best match (mzCloud or mzVault) score or 70 Fragment Ion Search (FISh) scoring was selected as a minimum threshold in selecting the candidate precursor ions in general.^42,48,49^ Finally, retention time model predictions were applied to the suspect compounds without available standards. Identification confidence was ranked through the Schymanski scale.^50^ In this study, level 1 refers to structural confirmation through reference standards. A level 2 confidence was assigned when the gap between modeled and experimental *RT*s (Δ*RT*) was ≤ 1.0 min. A Δ*RT* value above this threshold was denoted as level 3 confidence.

### QA/QC

The Orbitrap mass spectrometer was calibrated and evaluated weekly with the Pierce ESI negative ion calibration solution (Thermo Fisher Scientific, product no. 88324) through direct infusion. An eight-point external standard calibration consisting of 0.2, 0.5, 1, 2, 5, 10, 25, and 50 ng/mL mixed native standards (Table S1) was prepared in 50% methanol in water (0.1% acetic acid) with 5 ng/mL internal standards (Table S2). The linearity (*R* ^2^) of calibration curves was >0.99 for all standards within their calibration ranges. For every 6 randomly injected samples, at least one solvent blank was placed in the sequence to monitor carryover effects, along with one 10 ng/mL standard for instrument stability checks (i.e., peak intensity variation <15%; *RT* shift <0.1 min and mass error <5 ppm). The instrument detection limit (IDL, ng/mL in solvent) was estimated as the minimal analyte concentration at which the precursor ion signal-to-noise ratio (S/N) exceeded 3.^39^ Given the lack of standardized quantitative methods in NTA,^51,52^ calculating the method detection limit (MDL) remains challenging. For the purpose of assessing compound identification capability, we estimated the MDL (upper limit) as the lowest spiking level (1, 5, or 25 ng/mL in follicular fluid) when (1) the intensity of molecular ion signal was at least 3 times higher than that in the process blanks, and (2) a clear MS2 spectrum was generated in DDA. Method reproducibility was assessed through the relative standard deviation (RSD) of internal standard signal response in calibration standards (*n* = 8) and follicular fluid samples (*n* = 12). Processed in TraceFinder software (Thermo Fisher Scientific), detailed calculations on recoveries^53,54^ and matrix effects^55,56^ in spiked follicular fluid are included in Section 2 of the SI.

## Results and Discussion

### Fragmentation Patterns of Parabens and Phase I Metabolites

Broadly found in human biological samples, parabens are bioactive phenols with estrogenic impacts.^27,58^ However, most of the existing studies are heavily focused on the distribution of a few common parent species such as MeP, EtP, PrP, and BuP.^15,27^ To expand our knowledge of paraben identification in NTA, we first investigated the fragmentation pathways. For parent parabens, Figure 3a–e presents the MS/MS spectra of 5 representative standards (linear, branched, and benzyl parabens) at 20 eV CE, and Table S6 lists the distribution of major fragment ions at incremental CEs (10, 20, and 40 eV) for all species. Based on our experimental evidence, the proposed mechanisms are illustrated in Figure 4. Specifically, at low CEs (10 and 20 eV), fragmentation of paraben precursor ions is initiated by the heterolytic and homolytic cleavage of the R (alkyl or benzyl) group,^59^ producing 4-hydroxybenzoate ions at *m*/*z* 137.0239 (**A1**) and distonic ions at *m*/*z* 136.0160 (**A2**), respectively. Note that [R—H] loss is not structurally possible for MeP and BzP, and therefore, only **A2** is observed in Figure 3a,e. As CE increases, **A1** and **A2** further breakdown via α-elimination of CO_2_,^59,60^ forming phenolate ions at 93.0340 (**B1**) and *m*/*z* 92.0262 (**B2**). However, the intensity of **B2** tends to be significantly lower than that of **B1**, presumably due to its second radical (•H) loss as an intermediate species. The resulting fragment at *m*/*z* 91.0184 (**B3**) is known as a didehydrophenoxide biradical anion, as proposed by Schmidt et al.^61^ However, the continuous CO loss of **B3** indicated by Schmidt et al.^61^ was not observed in this study. To support the proposed structures of the product ions in Figure 4, we performed mass spectral fragmentation of an isotopically labeled standard (PrP-d4). The MS2 spectrum in Figure S3 shows identical deuterated 4-hydroxybenzoate ions and phenolate ions, the location of which may differ.

**Figure 3:**
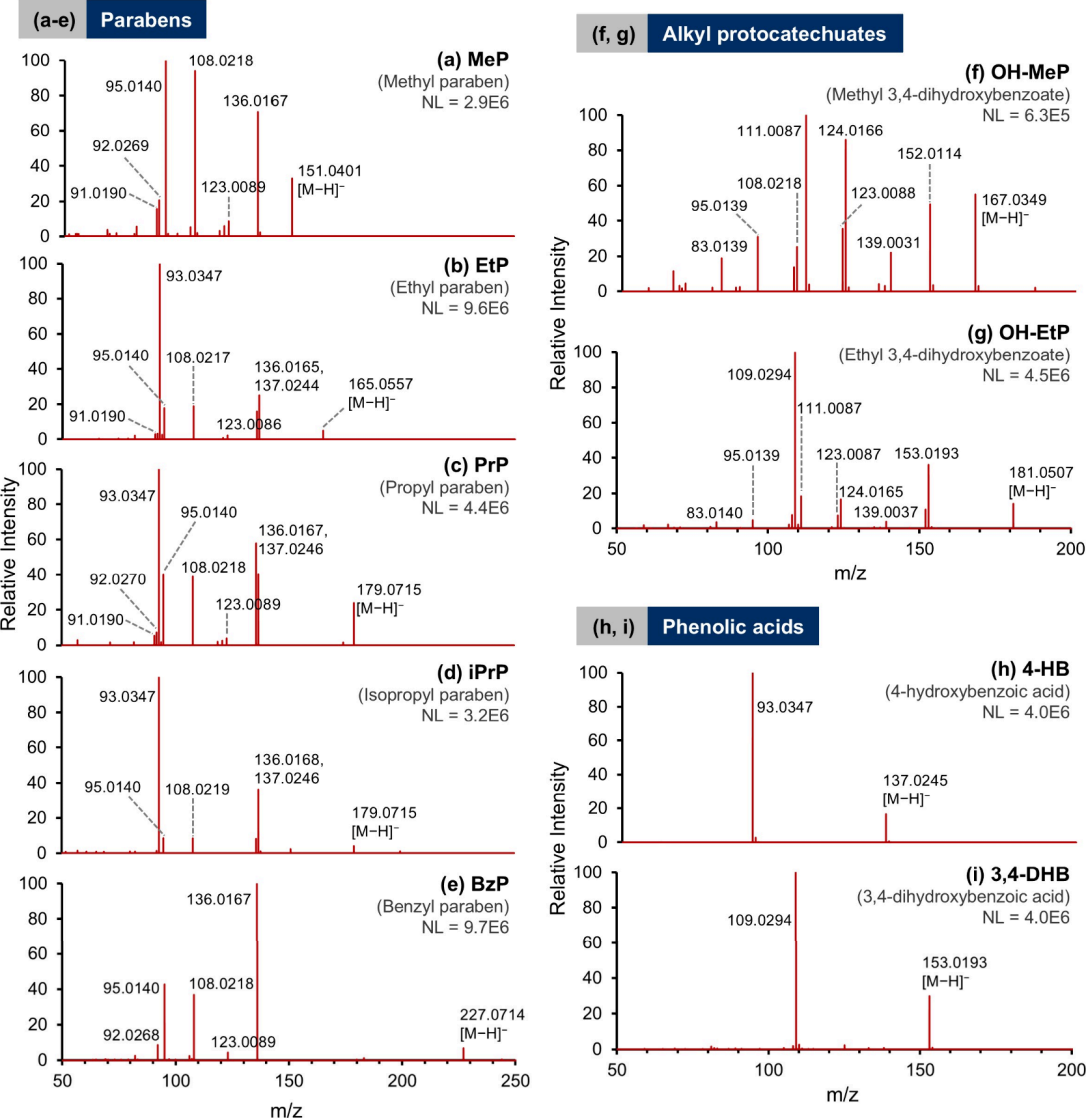
MS2 spectra of (a–e) selected parabens at 20 eV CE, (f, g) alkyl protocatechuates at 20 eV CE and (h, i) phenolic acids at 10 eV CE. The analyte concentration was 50 ng/mL, and the collision energies selected for the spectra shown were best conditions showing both precursor and fragment ions. See Table S6 for a complete list of fragment ions for all 14 standards at 10, 20, and 40 eV CEs. Abbreviations used in this work: **MeP** – methyl paraben; **EtP** – ethyl paraben; **PrP** – propyl paraben; **iPrP** – isopropyl paraben; **BzP** – benzyl paraben; **BuP** – butyl paraben; **iBuP** – **i**sobutyl paraben; **iPeP** – isopentyl paraben; **2-EtHeP** – 2-ethylhexyl paraben; **OcP** – octyl paraben; **4-HB** – 4-hydroxybenzoic acid; **3,4-DHB** – 3,4-dihydroxybenzoic acid; **OH–MeP** – methyl 3,4-dihydroxybenzoate; **OH-EtP** – ethyl 3,4-dihydroxybenzoate.

**Figure 4:**
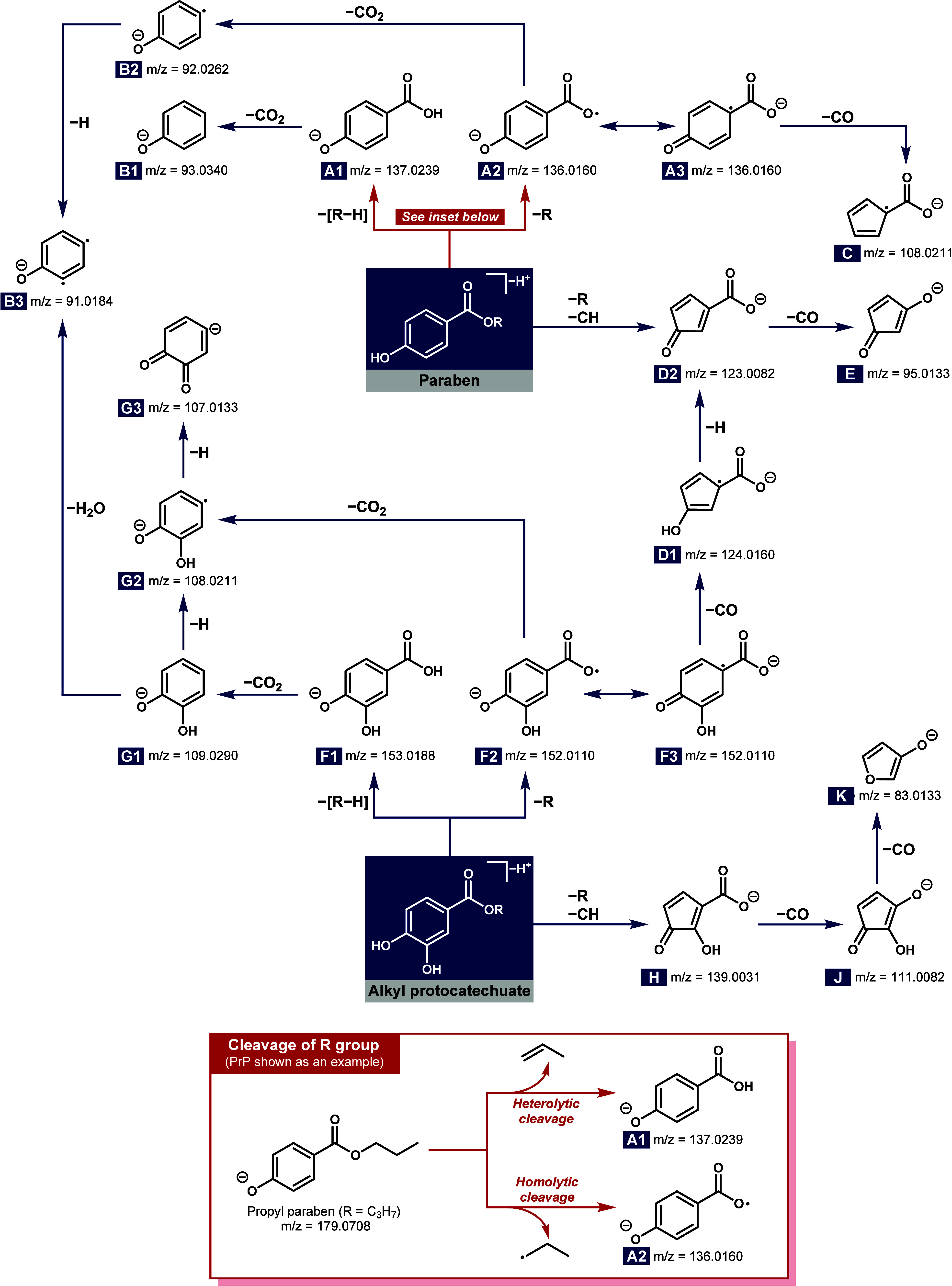
Proposed fragmentation pathways of parabens and alkyl protocatechuates. The listed mass-to-charge ratios correspond to the accurate monoisotopic masses (calculations via ChemCalc^57^).

Additional common fragmentation pathways for parabens are also observed in Figure 3a–e. For example, **A2** could transform into a resonance-stabilized cyclic carbonyl structure **A3**, which favors CO elimination that yields an ion at *m*/*z* 108.0211 (**C**).^60,62^ In addition, the product ion at *m*/*z* 123.0082 (**D2**) corresponds to **A2** with an additional CH loss. Its proposed structure is supported by the observation of a deuterium loss from the aromatic ring in PrP-d4 (*m*/*z* = 126.0276 in Figure S3). A subsequent CO elimination produces an ion at *m*/*z* 95.0133 (**E**),^63^ which was detected in the spectra of all parabens in this work. Overall, we identified 8 characteristic product ions that can be used as diagnostic ions for screening parabens. This observed fragmentation mechanism complements the previous paraben measurements involving triple-quadrupole (TQ) and quadrupole time-of-flight (qTOF) mass spectrometers. Specifically, only *m*/*z* 92, 93, 136, and 137 have been reported as fragments in multiple reaction monitoring (MRM) transitions for targeted analysis,^64–67^ and a recent non-targeted workflow developed by Chi et al. was limited to two diagnostic ions (*m*/*z* 92 and 93).^68^ In our present work, while we also observed these fragments starting at 10 eV CE, a higher CE (20 or 40 eV) facilitated further dissociation, yielding product ions at *m*/*z* 91, 95, 108, and 123. This highlights the differences between traditional low-energy collision-induced dissociation (CID) and higher-energy collisional dissociation (HCD) pathways in Orbitrap mass spectrometers. Similar distinctions between CID and HCD spectra have been recorded in proteomic identifications.^69,70^ Moreover, we also matched the experimental MS2 spectra in Figure 3a–e with those published in the mzCloud library and the simulated spectra generated by the *in silico* algorithm (Mass Frontier). At the time of writing, the online database had limited coverage of paraben species acquired in the negative ESI mode, while only two common ions at *m*/*z* 92 and 136 were matched by the simulator (an example of MeP shown in Figure S4). Such discrepancies have been noted in other recent evaluations.^40,41^ Thus, we expect that the inclusion of experimentally confirmed diagnostic ions would significantly enhance the annotation confidence of parabens.

As a part of phase I metabolism (see Figure 1, upper section), hydroxylation is an important reactive pathway for parabens absorbed by the human body.^27,28^ The resulting products, alkyl protocatechuates (also known as 3,4-dihydroxybenzoates), have been identified as urinary biomarkers for paraben exposure analyses.^27,28,58^ In fact, higher concentrations of alkyl protocatechuates have been reported in urine than their respective parent parabens.^28^ However, biomonitoring studies on hydroxylated metabolites are limited to OH–MeP and OH-EtP due to the availability of reference materials, potentially underestimating the total cumulative paraben exposure. Inspired by the identification of diagnostic fragmentation pathways of parabens in this work, we also hypothesized that alkyl protocatechuates would follow similar patterns, with a unique set of product ions. To test this, tandem MS was performed on OH–MeP and OH-EtP (i.e., the only available standards). As expected (Figure 3f,g), the fragmentation mechanisms follow the patterns of parabens as many observed ions contained an additional oxygen (Δ*m*/*z* = 15.9949). Notably, as proposed in Figure 4, the product ions at *m*/*z* 153.0188 (**F1**) and/or 152.0110 (**F2**) undergo a decarboxylation process to form 2-hydroxyphenolate ions at *m*/*z* 109.0290 (**G1**), 108.0211 (**G2**), and 107.0133 (**G3**). Furthermore, similar to the observed ion **D2** and **E** in parabens, a single and double neutral loss of CO from *m*/*z* 139.0031 (**H**) yield ions at *m*/*z* 111.0082 (**J**) and 83.0133 (**K**), respectively.

It is noteworthy that some identical characteristic ions were identified in both paraben parents and alkyl protocatechuate metabolites. Specifically, the ion at *m*/*z* 124.0160 (**D1**), formed via a neutral CO elimination from **F3**, is proposed to undergo successive losses of a hydrogen radical and a CO,^60,62^ yielding ions at *m*/*z* 123.0082 (**D2**) and *m*/*z* 95.0133 (**E**). Additionally, the characteristic ion at *m*/*z* 91.0184 (**B3**) is attributable to a H_2_O loss from **G1**. In fact, from a metabolomics perspective, this fragmentation pattern provides important structural and metabolism site^71^ information as it confirms hydroxylation occurring on the aromatic ring. Lastly, to include all phase I paraben metabolites, we also investigated the fragmentation pathways of phenolic acids. For 4-HB (precursor ion identical to **A1**), only *m*/*z* 93.0340 (**B1**) was observed at 10, 20, and 40 eV CEs (Figure 3h and Table S6), and the absence of **B2** and **B3** in the observed spectra indicates that •H elimination is not feasible for **B1**. Conversely, a loss of CO_2_ from the deprotonated 3,4-DHB (**F1**) produces *m*/*z* 109.0290 (**G1**) at 10 eV CE (Figure 3i). However, at 20 and 40 eV CEs (Table S6), it is subject to further •H or H_2_O elimination that yields **G2** and **B3**. In summary, we have identified a total of 17 common fragment ions covering paraben parents and their phase I metabolites through tandem MS of 14 standards. It should be noted that phase II metabolites (Figure 1, lower section) were not investigated in this manner due to the lack of reference standards. However, the diagnostic fragment ions described in Figure 4 are still useful for identifying paraben conjugates (vide infra).

### Compound Identification Using Diagnostic Ions and Compound Discoverer in Spiked Follicular Fluid

To evaluate the workflow performance, we first assessed the analytical sensitivity via detection limit estimations of compounds within the chemical space. Specifically, as listed in Table S7, 60% of the 64 EDC standards had IDLs below the lowest calibrant concentration (0.2 ng/mL), while the detection of some bisphenols tended to be much less sensitive. This was not surprising as bisphenols are well-known for their poor ionization efficiency in negative ESI, which requires mobile phase additives with high gas-phase proton affinity.^72^ In the follicular fluid sample matrix, approximately 40% and 75% of the analytes were reliably detected with abundant precursor ion intensities and clear MS2 spectra in ddMS2 (as a measure of MDL estimation), at 1 and 5 ng/mL spiking levels, respectively. A few compounds, such as 2,4-dichlorophenol and 2,4,6-trichlorophenol, showed low IDLs but high MDLs, likely due to possible procedural loss during extraction. Varying from 12 to 20%, method reproducibility was calculated through the RSD of precursor peak areas of the internal standards added in follicular fluid samples (Table S8). In terms of accuracy,^53^ the recovery range was between 75 and 125% for approximately 30, 60, and 70% of the analytes at the 1, 5, and 25 ng/mL spiking levels, respectively (Table S9). Lastly, while thorough cleanup steps were performed during sample treatment, the sample matrix still caused slight to moderate signal suppression (10–30%) for most analytes based on matrix effect (ME) calculations (Table S9). This could be due to coeluting ions (including residual matrix ions) competing for the limited capacity of the C-trap.

After identifying the recovered EDCs in spiked follicular fluid by their precursor ions, we then assessed the feasibility of compound identification based on fragmentation patterns. First, with the use of diagnostic paraben fragment ions characterized in the previous section, Figure 5 shows the extracted ion chromatograms (EICs) of these ions in the MS2 spectra of the spiked sample (25 ng/mL). Overall, both DDA (Figure 5a–g) and DIA (Figure 5h–n) acquisition modes were able to demonstrate the presence of spiked parabens and metabolites, but some variations were observed. Specifically, as seen in the EICs of diagnostic ions for both parabens and alkyl protocatechuate (Figure 5a–c,h–j), while the DIA spectra illustrate smoother peaks with higher peak intensity, DDA results are more informative. For example, coeluting compounds (MeP and OH-EtP; BzP and iPeP) are more easily identified in DDA due to its direct selection of precursor ions. Similar differences between the two modes have been previously reported in NTA of monophthalates.^38^

**Figure 5:**
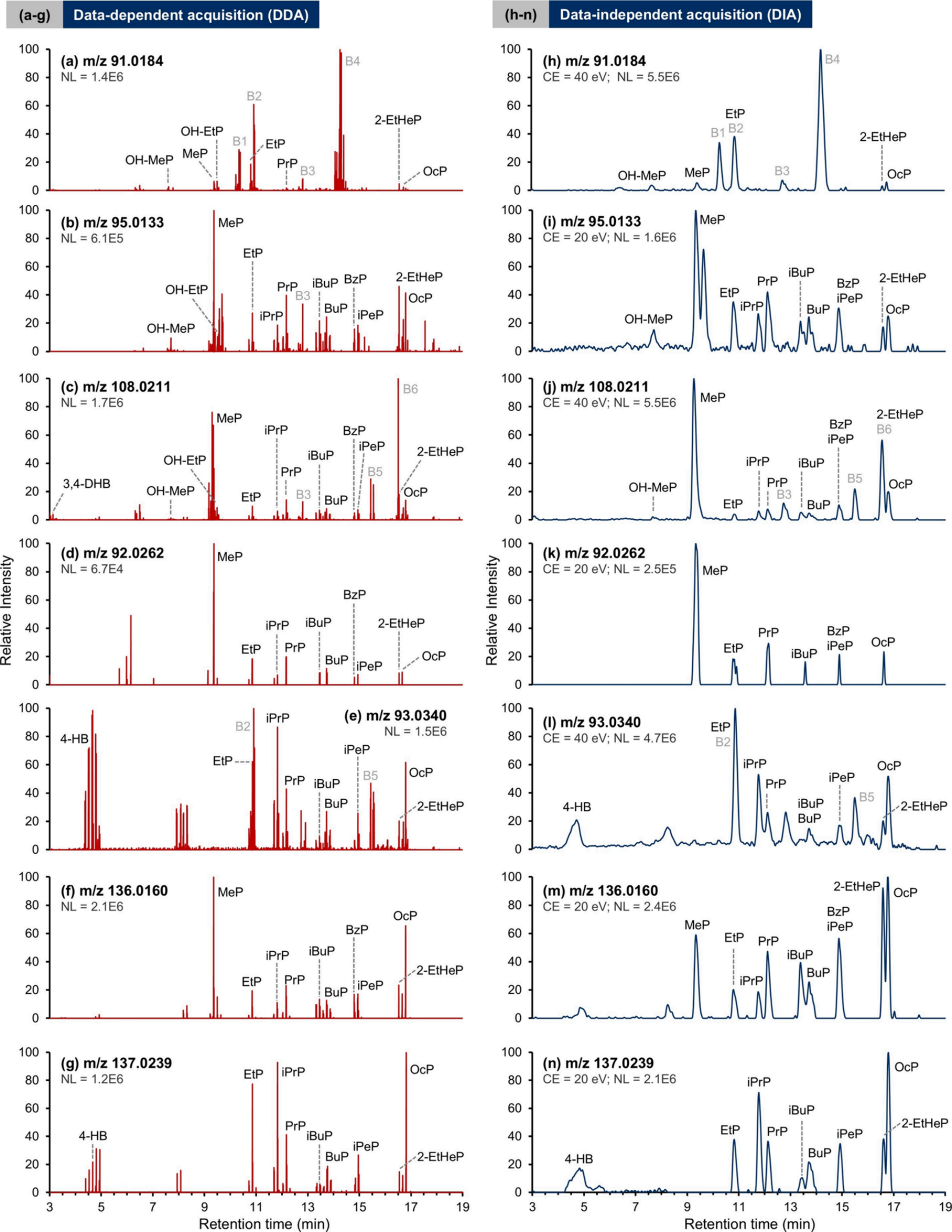
Extracted ion chromatograms (EICs) of paraben diagnostic fragment ions in (a–g) DDA and (h–n) DIA data of a pooled follicular fluid sample with a spiking concentration of 25 ng/mL. The diagnostic ion at *m*/*z* 123.0082 is not displayed due to its relatively weak peak intensity. The chromatograms shown in DDA correspond to a combination of spectra collected at 10, 20, and 40 eV CEs, while for DIA, a preferred CE (20 or 40 eV) was chosen to demonstrate the best data quality. B1–B6 correspond to the spiked benzophenone standards: B1 – benzophenone-2; B2 – 2,4,4 trihydroxybenzophenone; B3 – 4-hydroxybenzophenone; B4 – benzophenone-1; B5 – dioxybenzone; and B6 – benzophenone-6.

It is noteworthy that strong interfering peaks (denoted as B1–B6) were found in the EICs of the diagnostic ions discussed above, especially for *m*/*z* 91. Confirmed with the fragmentation profiles of pure standards, these peaks resulted from benzophenones that were among the 64 EDCs in the spiked samples. Thus, to minimize the effects of other interfering phenolic compounds, the inclusion of a fourth (or more) diagnostic ion could reduce the chance of false positives. Accordingly, Figure 5d–g,k–n shows EICs of additional diagnostic ions for parabens, and similarly, DDA spectra were more informative, especially for illustrating weak signals such as *m*/*z* 92 (Figure 5d,k). Moreover, EICs of *m*/*z* 136 and 137 had the cleanest background for this follicular fluid matrix (Figure 5f,g,m,n). Figure S5 compares DDA and DIA in spiked samples at 1 ng/mL, and it is not surprising that fewer peaks were observed in both modes. This poses a challenge for precursor ion isolation in DIA data, since the spectral background significantly interferes with the diagnostic ions in EICs. Comparatively, although DIA can be effective for identifying a higher number of analytes,^22,34,37^ our DDA data exhibited sufficient sensitivity for paraben identification when four diagnostic fragment ions were set as a threshold for prioritizing precursors.

Given that not all EDC chemicals can be traced to a known diagnostic fragmentation pattern, we then evaluated compound annotation for a wider range of EDCs through mass spectral library matching in Compound Discoverer, including the use of an in-house database built in this work and *in silico* fragmentation. With 70 as the threshold matching score, the true positive identification rates, defined as the proportion of matched compounds out of 64 spiked EDCs,^73^ were 41, 70, and 83% for samples with 1, 5, and 25 ng/mL spiking levels, respectively (Table S10). This result is similar to our previous work on chemical identification in spiked urine (∼50% rate at 1 ng/mL spiking level).^39^ A common issue for extracted precursor ions not being identified through library matching was the failure of triggering fragmentation in DDA scans.^38,74^ This was due to their signal intensity near or below the preset threshold value. To improve the chances of analyte selection and fragmentation, we attempted to adjust scan parameters such as lowering the signal intensity threshold to 1.5E5 (via reducing the minimum AGC target to 8.0E3) and increasing the loop count (or topN) to 6, 8, and 10. However, from our observations, more coeluting matrix ions entered the C-trap simultaneously for scanning in the Orbitrap, thereby increasing the spectral noise and lowering the overall compound matching scores.

### NTA Application in Nonspiked Follicular Fluid

The newly developed NTA workflow was applied to nonspiked follicular fluid samples, with and without enzymatic treatment, to assess its capability for identifying unknown compounds. Here, 500 μL pooled samples were processed (see Section 2 of SI) to facilitate the detection of ultralow-concentration species. In hydrolyzed samples, based on the selection criterion of at least four diagnostic fragment ions in DDA data, 5 candidate precursor ions were identified as parabens (MeP, EtP, PrP) and protocatechuates (OH–MeP and OH-EtP). Together with their metabolized acids (4-HB and 3,4-DHB), all compounds were annotated with level 1 confidence (Schymanski scale^50^) after retention time confirmation with standards (Table S11). Tentatively assigned as level 3, several positional isomers of these species, which share identical MS and MS2 profiles but differ in retention times, were also proposed. The use of the Compound Discoverer further enabled structural deconvolution of some suspect compounds, as discussed later in this section.

The extraction of paraben diagnostic ions also allowed us to identify the speciation of phase II metabolites present in unhydrolyzed follicular fluid. As demonstrated in Figure 6a–f, six precursor ions were identified as sulfate conjugates of parabens and their phase I metabolites, with detailed fragmentation profiles provided in Table S12. This identification was supported by the formation of the SO_3_^—^ ion at *m*/*z* 80,^75^ yielding deprotonated ions of free parabens (or their phase I metabolites) that continued to fragment via their diagnostic pathways. In some cases, the HSO_4_^–^ ion at *m*/*z* 97 was also observed.^75^ The measured *RT*s of the proposed conjugates were compared against the model prediction or standards (Table S12). Notably, five of six identified metabolites were assigned level 2 confidence (Δ*RT* ≤ 0.5 min), while PrP sulfate was further confirmed using its standard, reaching level 1 confidence. Conversely, only 3,4-DHB sulfate was assigned level 3 confidence, owing to a larger discrepancy between the measured and predicted *RT*s.

**Figure 6:**
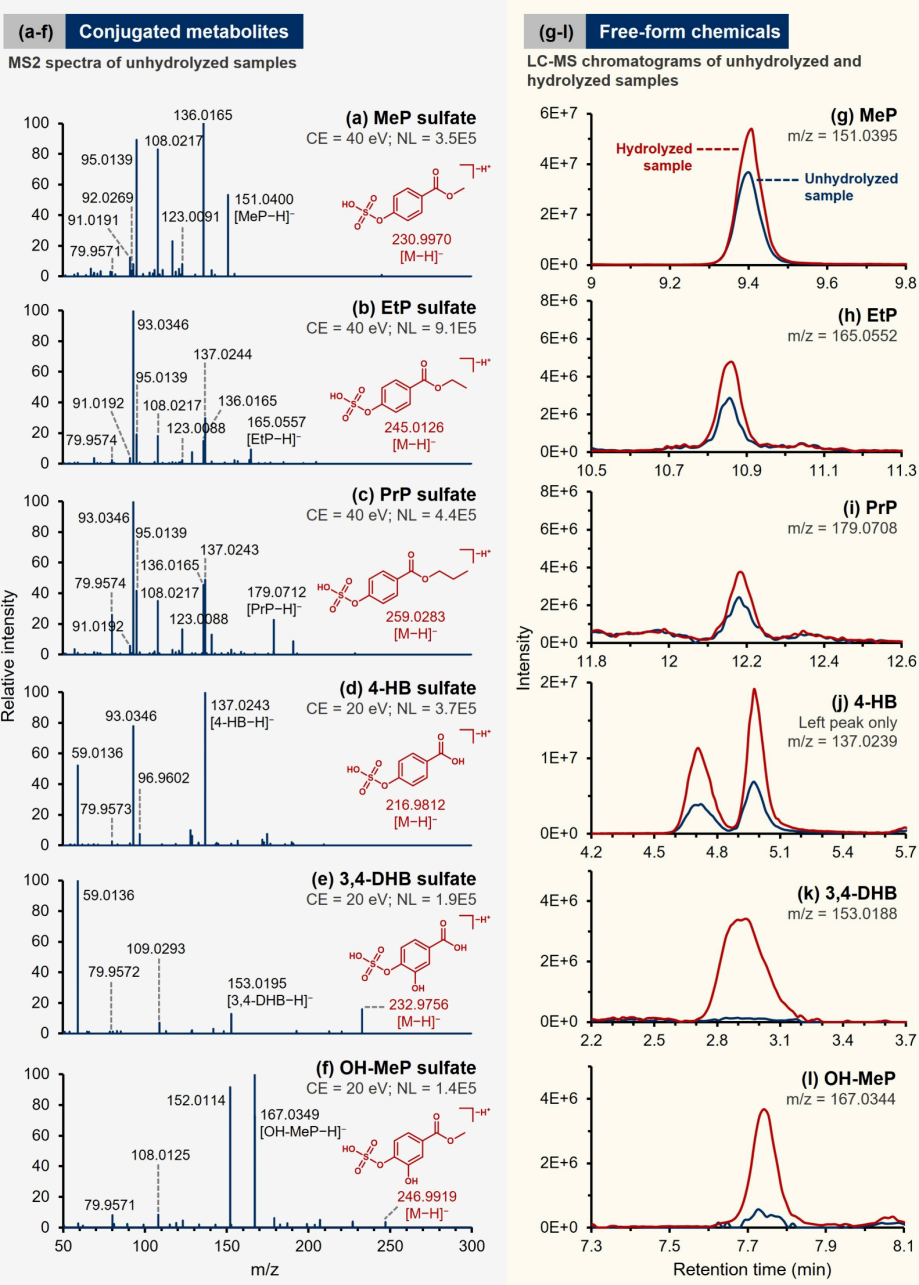
(Left side) MS2 profiles of proposed phase II paraben metabolites (sulfates) identified in unhydrolyzed pooled follicular fluid samples: (a) methyl paraben sulfate, (b) ethyl paraben sulfate, (c) propyl paraben sulfate, (d) 4-hydroxybenzoic acid sulfate, (e) 3,4-dihydroxybenzoic acid sulfate, and (f) methyl 3,4-dihydroxybenzoate sulfate. (Right side) Extracted ion chromatograms (EICs) of corresponding free-from chemicals (precursor ions with mass error ± 5 ppm) in hydrolyzed and unhydrolyzed follicular fluid samples: (g) methyl paraben, (h) ethyl paraben, (i) propyl paraben, (j) 4-hydroxybenzoic acid, (k) 3,4-dihydroxybenzoic acid, and (l) methyl 3,4-dihydroxybenzoate.

More importantly, given that none of the proposed sulfate conjugates was detected in hydrolyzed samples, a comparison of free-from chemicals between hydrolyzed and unhydrolyzed samples can reveal the distribution of paraben speciation in follicular fluid. Accordingly, from the EIC chromatograms in Figure 6g–l, while free parabens such as MeP and EtP were detected in unhydrolyzed samples, enzymatic hydrolysis increased their intensity. In particular, 3,4-DHB and OH–MeP (Figure 6k,l) appeared to exist predominantly in their conjugate forms in the original fluid. Without any evidence of detecting paraben-based glucuronides in this study, we thus infer that sulfation is likely a dominant phase II metabolism for EDCs of this type. Although conjugated parabens have long been reported in biological matrices,^76^ detailed structural evidence of individual species remains rare. Our results complement the recent NTA works that also suggest the significance of sulfation metabolism, as shown through urine^63^ and human milk analyses.^68^

Finally, more compounds were identified through mass spectral library matching in Compound Discoverer. In the analysis of hydrolyzed samples, the software detected more than 6000 molecular features, the vast majority of which had only exact mass information without MS2 spectral matches, corresponding to level 4–5 confidence.^50^ Within our chemical space, we selected 37 features based on a minimum match score of 70 (mzCloud, mzVault, or FISh), allowing structure annotations at level 3 confidence or higher. A detailed summary is listed in Table S13. Briefly, a total of 14 features were identified as level 1 confidence. Apart from parabens and metabolites mentioned above in Table S11, monophthalates (monomethyl-, monoethyl-, and monoisobutyl phthalate), UV filters (benzophenone-1) and metabolized organic acids were among the other potential EDCs found in this pooled follicular fluid sample. One compound, initially proposed as a 4-HB isomer (*RT* = 8.1 min) through diagnostic fragmentation ions (Table S11), was later confirmed to be salicylic acid (2-HB) through database search and *RT* match with a standard. Notably, Chen et al. detected elevated levels of salicylic acid in follicular fluid that were four times higher in patients with polycystic ovary syndrome (PCOS) than in the healthy control group,^77^ due to its association with phenylalanine metabolism that affects oocyte quality.^77,78^ As well, hippuric acid, a glycine conjugate of benzoic acid used as a biomarker for metabolic health^79^ and toluene,^80^ was also annotated as level 1. Lastly, as explained in Figure S6, three molecular features matched the MS2 fragmentation patterns of 4-hydroxyhippuric acid (4-HHA, structure shown in Figure 1), an amino acid derivative of 4-HB. Further retention time validation using a pure standard confirmed level 1 identification for the precursor ion eluting at 3.1 min. Thus, the use of library search facilitated the identification of another potential phase II paraben metabolite,^29,58^ whose MS2 fragmentation pattern differs from that of the parent parabens. Although Compound Discoverer suggested a match to 2-hydroxyhippuric acid (2-HHA) for the other two features shown in Figure S6, this identification was not supported by the *RT* model prediction (Table S13) for assignments at level 2 confidence.

## Conclusions

EDCs in follicular fluid are important indicators in evaluating oocyte quality and reproductive health,^18,19^ and thus, a robust analytical method detecting a wide range of species is valuable for biomonitoring studies. In the present work, we developed a new non-targeted workflow consisting of sample treatment, data acquisition, and data analysis to meet this need. Our experimental results demonstrated that parabens (and protocatechuates) followed a characteristic fragmentation pattern, yielding unique diagnostic ions in HCD cells. With high sensitivity and precision, the extraction of such mass spectral fingerprints enables rapid prioritization of paraben precursor ions in DDA. Conversely, DIA requires complex deconvolution for interpretation at this point, but ongoing development of computational tools could enhance its capabilities in the future.^81^ More importantly, our paraben annotation approach is also much more reliable than database matching through online library and *in silico* fragmentation. In particular, we examined that the latter algorithm led to high errors in predicting the MS2 spectra, an observation consistent with the recent criticisms calling for cautious use of simulator tools.^40,41^ Nevertheless, the application of diagnostic evidence is still limited to specific groups of chemicals, and broader compound identifications through spectral database matching remain an important aspect of NTA. In this work, species of potential concern, such as monophthalates, UV filters, and metabolized organic acids, were confirmed in follicular fluid, highlighting the utility of this method for identifying EDC bioaccumulation linked to human exposure.

This NTA work not only revealed the distribution of exogenous substances in human biological samples but also implied the possible metabolic processes occurring in the human body. To the best of our knowledge, this is the first study that identifies parabens, as well as their phase I and phase II metabolites, simultaneously in follicular fluid samples. Specifically, hydrolysis and hydroxylation products were identified and confirmed using standards. Annotated at level 1 and 2 confidence, sulfate and glycine conjugates were the detected forms of phase II metabolic biomarkers for parabens. These conjugates have rarely been characterized in biological samples due to the limited availability of their reference standards. In this work, although no firm evidence was found for paraben glucuronidation, the ability to screen such large molecules through diagnostic fragment ions improves our understanding of metabolite speciation for more accurate exposure assessments.

Lastly, some limitations remain in the workflow and are worth further research attention. First of all, the present method was designed for extracting and detecting phenolic compounds and metabolized organic acids; its analysis capacity over other major groups of EDCs (e.g., polychlorinated biphenyls)^3^ remains to be validated. Within the chemical space, while high identification rates were attained for compounds such as parabens and monophthalates, along with satisfactory recoveries and reproducibility, the current method underperformed in extracting and identifying (1) bisphenols and halogenated phenols and (2) some EDCs with ultralow concentrations (i.e., ≤1 ng/mL). Thus, inspired by recent comprehensive optimization works on Orbitrap instruments,^74,82^ our future work will focus on sensitivity improvements in NTA data acquisition. Furthermore, the present NTA workflow is limited to qualitative identification in follicular fluid. Similar to our in-house retention time model, future improvements on quantitative non-targeted analysis (qNTA) can be developed through modeling with the molecular descriptors.^83,84^ Such quantitative data from individual patient analyses, obtained through both targeted analysis and qNTA, will help us determine any potential correlations between chemical exposure and infertility conditions.

## Supplementary Material



## Notes

List of standards and internal standards, method descriptions (treatment of 500 μL pooled follicular fluid samples, retention time prediction model, recovery and matrix effect calculations, and key Compound Discoverer settings), diagnostic fragmentation of paraben ions, compound identification in spiked follicular fluid, and compound identification in nonspiked follicular fluid (PDF)

## ^

Afynia Laboratories, Hamilton, ON L8P 0A6, Canada (W.G.F.)
