# Supplementary material for: Non-Targeted Analysis Workflow of Endocrine-Disrupting Chemicals in Ovarian Follicular Fluid: Identification of Parabens by Diagnostic Fragmentation Evidence and Additional Contaminants via Mass Spectral Library Matching
Source: ACS Meas Sci Au. 2025 Aug 21;5(6):790–804. doi: 10.1021/acsmeasuresciau.5c00082 (PMC12715743; doi:10.1021/acsmeasuresciau.5c00082)
Supplement: Supplementary file 1 [file tg5c00082_si_001.pdf]

Supporting Information

Non-targeted analysis workflow of endocrine-disrupting chemicals in ovarian follicular fluid: identification of parabens by diagnostic fragmentation evidence and additional contaminants via mass spectral library matching

Zilin Zhou[1], Michael S. Neal,[2,3] Warren G. Foster[3^] and Yong-Lai Feng[1]*

[1] Exposure and Biomonitoring Division, Environmental Health Science and Research Bureau, Environmental and Radiation Health Sciences Directorate, Healthy Environments and Consumer Safety Branch, Health Canada, Ottawa, ON K1A 0K9, Canada

[2] ONE Fertility, Burlington, ON L7N 3T1, Canada

[3] Department of Obstetrics & Gynecology, McMaster University, Hamilton, ON L8S 4L8, Canada

[^] Present address: Afynia Laboratories, Hamilton, ON L8P 0A6, Canada

* Corresponding author. Email: yong-lai.feng@hc-sc.gc.ca

This 41-page document contains 5 sections, including 13 tables and 6 figures.

Table of Contents

1. Standards and internal standards

Table S1. Native standards used as spiking chemicals in follicular fluid samples

Table S2. Isotopic labelled standards in calibration curves, processed blanks and follicular fluid samples

Table S3. Additional standards used in retention time development and structural confirmation in NTA

2. Methods

Treatment of 500 µL pooled follicular fluid samples

Retention time prediction model

Table S4. Constant, coefficients, and molecular descriptors used in the retention time model equation

Figure S1. Comparison of experimental and predicted retention times

Table S5. Detailed retention time comparison in the testing set

Recovery and matrix effect calculations

Key Compound Discoverer settings

Figure S2. Compound Discoverer workflow tree in this study

3. Diagnostic fragmentation of paraben ions

Figure S3. MS/MS spectra of 50 ng/mL n-propyl 4-hydroxybenzoate-2,3,5,6-d4

Table S6. Precursor ions and major product ions of paraben standards and their phase I metabolites

Figure S4. Experimental and predicted tandem MS/MS spectra of methyl paraben

4. Compound identification in spiked follicular fluid

Table S7. Measured information of EDC standards spiked in follicular fluid samples

Table S8. Measured information of internal standards spiked in the pooled follicular fluid samples

Table S9. Recoveries and matrix effect of EDC standards in the spiked 100 µL follicular fluid samples

Table S10. Summary of EDC standard identification in three spiking levels of follicular fluid samples

Figure S5. Extracted ion chromatograms (EICs) of paraben diagnostic fragmentation ions (1 ng/mL)

5. Compound identification in non-spiked follicular fluid

Table S11. Parabens and phase I metabolites identified in hydrolyzed pooled follicular fluid

Table S12. Phase II metabolites of parabens identified in unhydrolyzed pooled follicular fluid

Table S13. Phenols and metabolized organic acids identified in hydrolyzed follicular fluid

Figure S6. Extracted ion chromatograms (EICs) and MS2 spectra for 4-hydroxyhippuric acid identification

References

1. Standards and internal standards

Table S1. (3 pages) 64 native standards used as spiking chemicals in follicular fluid samples.

Analyte	Molecular formula	CAS	MW (g/mol)	Purity/ Grade	Supplier
			**Mono-phthalates**		
**MHBP** [Mono (3-hydroxybutyl) phthalate]	$C_{12}H_{14}O_5$	57074-43-8	238.24	≥95%	TRC
Mono (3-carboxypropyl) phthalate	$C_{12}H_{12}O_6$	66851-46-5	252.22	≥97%	TRC
**MMP** (Monomethyl phthalate)	$C_9H_8O_4$	4376-18-5	180.16	≥99%	Sigma Aldrich
Monomethyl isophthalate	$C_9H_8O_4$	1877-71-0	180.16	≥98%	Sigma Aldrich
**MEP** (Monoethyl phthalate)	$C_{10}H_{10}O_4$	2306-33-4	194.18	≥98%	TRC
Monoisopropyl phthalate	$C_{11}H_{12}O_4$	35118-50-4	208.21	≥97%	TRC
**MBP** (Monobutyl phthalate)	$C_{12}H_{14}O_4$	131-70-4	222.24	≥99%	Sigma Aldrich
**MIBP** (Monoisobutyl phthalate)	$C_{12}H_{14}O_4$	30833-53-5	222.24	≥98%	TRC
Mono(carboxyisooctyl) phthalate	$C_{17}H_{22}O_6$	898544-09-7	322.40	≥97%	Santa Cruz Technologies
Mono-2-carboxy-methylhexyl phthalate	$C_{16}H_{20}O_6$	82975-93-7	308.33	≥90%	TRC
**MBzP** (Monobenzyl phthalate)	$C_{15}H_{12}O_4$	2528-16-7	256.25	≥99%	TRC
**MCHP** (Monocyclohexyl phthalate)	$C_{14}H_{16}O_4$	7517-36-4	248.27	≥98%	TRC
Mono-n-pentyl phthalate	$C_{13}H_{16}O_4$	24539-56-8	236.26	≥98%	TRC
Mono-9-carboxynonyl phthalate	$C_{18}H_{24}O_6$	-	336.38	≥98%	TRC
Monohexyl phthalate	$C_{14}H_{18}O_4$	24539-57-9	250.29	Not specified	CanSyn
Monoheptyl phthalate	$C_{15}H_{20}O_4$	24539-58-0	264.32	≥99%	TRC
Monooctyl phthalate	$C_{16}H_{22}O_4$	5393-19-1	278.34	≥97%	TRC
Mono-5-carboxypentyl phthalate	$C_{14}H_{16}O_6$	92569-49-8	280.27	≥98%	TRC
Monopropyl phthalate	$C_{11}H_{12}O_4$	4376-19-6	208.21	≥97%	TRC
**MEHP** [Mono (2-ethylhexyl) phthalate]	$C_{16}H_{22}O_4$	4376-20-9	278.34	≥95%	TRC
			**Parabens and metabolites**		
**MeP** (Methyl paraben)	$C_8H_8O_3$	99-76-3	152.15	≥98%	Sigma Aldrich
**EtP** (Ethyl paraben)	$C_9H_{10}O_3$	120-47-8	166.17	≥98%	Sigma Aldrich

**PrP** (Propyl paraben)	$C_{10}H_{12}O_3$	94-13-3	180.20	≥99%	Sigma Aldrich
**BzP** (Benzyl paraben)	$C_{14}H_{12}O_3$	94-18-8	228.24	≥98%	TCI
**BuP** (Butyl paraben)	$C_{11}H_{14}O_3$	94-26-8	194.23	≥98%	Spectrum Chemical
**iPeP** (Isopentyl paraben)	$C_{12}H_{16}O_3$	6521-30-8	208.26	≥98%	TCI
**2-EtHeP** (2-ethylhexyl paraben)	$C_{15}H_{22}O_3$	5153-25-3	250.34	≥98%	TCI
**iBuP** (Isobutyl paraben)	$C_{11}H_{14}O_3$	4247-02-3	194.23	≥99%	TCI
**iPrP** (Isopropyl paraben)	$C_{10}H_{12}O_3$	4191-73-5	180.20	≥99%	TCI
**OcP** (n-Octyl paraben)	$C_{15}H_{22}O_3$	1219-38-1	250.34	≥98%	Thermo Scientific Chemicals
**4-HB** (4-Hydroxy benzoic acid)	$C_7H_6O_3$	99-96-7	138.12	≥99%	Sigma Aldrich
**3,4-DHB** (3,4-Dihydroxybenzoic acid)	$C_7H_6O_4$	99-50-3	154.12	≥97%	Thermo Scientific Chemicals
**OH-MeP** (Methyl 3,4-dihydroxybenzoate)	$C_8H_8O_4$	2150-43-8	168.15	≥98%	TCI
**OH-EtP** (Ethyl 3,4-dihydroxybenzoate)	$C_9H_{10}O_4$	3943-89-3	182.17	≥97%	TRC
**Phenols**					
**BPA** (Bisphenol A)	$C_{15}H_{16}O_2$	80-05-7	228.29	≥99%	Sigma Aldrich
**BPB** (Bisphenol B)	$C_{16}H_{18}O_2$	77-40-7	242.31	≥98%	Sigma Aldrich
**BPS** (Bisphenol S)	$C_{12}H_{10}O_4S$	80-09-1	250.27	≥99%	Sigma Aldrich
**TDP** (4,4'-Thiodiphenol)	$C_{12}H_{10}O_2S$	2664-63-3	218.27	≥99%	Sigma Aldrich
Bisphenol TMC	$C_{21}H_{26}O_2$	129188-99-4	310.43	≥99%	AccuStandard
**BHPF** (Bisphenol FL)	$C_{25}H_{18}O_2$	3236-71-3	350.41	≥99%	Sigma Aldrich
**BPG** (Bisphenol G)	$C_{21}H_{28}O_2$	127-54-8	312.45	≥98%	Sigma Aldrich
Bisphenol BP	$C_{25}H_{20}O_2$	1844-01-5	352.43	≥98%	Sigma Aldrich
Bisphenol PH	$C_{27}H_{24}O_2$	24038-68-4	380.48	≥99%	Sigma Aldrich
**BPM** (Bisphenol M)	$C_{24}H_{26}O_2$	13595-25-0	346.46	≥99%	Sigma Aldrich
**BPP** (Bisphenol P)	$C_{24}H_{26}O_2$	2167-51-3	346.46	≥99%	Sigma Aldrich
4-Phenylphenol	$C_{12}H_{10}O$	92-69-3	170.21	≥99%	Sigma Aldrich
**Halogenated phenols**					
Dichlorophen	$C_{13}H_{10}Cl_2O_2$	97-23-4	269.12	Analytical standard grade	Sigma Aldrich
Hexachlorophen	$C_{13}H_6Cl_6O_2$	70-30-4	406.90	Analytical standard grade	Sigma Aldrich
Bithionol	$C_{12}H_6Cl_4O_2S$	97-18-7	356.05	Analytical standard grade	Sigma Aldrich

**TBBPA** (3,3',5,5',-Tetrabromobisphenol A)	$C_{15}H_{12}Br_4O_2$	79-94-7	543.87	Analytical standard grade	Sigma Aldrich
**TBBPS** (Tetrabromobisphenol S)	$C_{12}H_6Br_4O_4S$	39635-79-5	565.85	≥95%	TRC
Triclosan	$C_{12}H_7Cl_3O_2$	3380-34-5	289.54	Certified reference material	Sigma Aldrich
2,4-Dichlorophenol	$C_6H_4Cl_2O$	120-83-2	163.00	≥99%	Sigma Aldrich
2,4,6-Trichlorophenol	$C_6H_3Cl_3O$	88-06-2	197.45	≥98%	Sigma Aldrich
2,4-Dibromophenol	$C_6H_4Br_2O$	615-58-7	251.90	≥95%	Sigma Aldrich
2,4,6-Tribromophenol	$C_6H_3Br_3O$	118-79-6	330.80	≥95%	Sigma Aldrich
4,6-Dichlororesorcinol	$C_6H_4Cl_2O_2$	137-19-9	179.00	≥98%	TRC
Triclocarban	$C_{13}H_9Cl_3N_2O$	101-20-2	315.58	≥98%	TRC
**UV filters**					
**Benzophenone-2** (2,2,4,4 Tetrayhydroxy-benzophenone)	$C_{13}H_{10}O_5$	131-55-5	246.22	≥97%	Sigma Aldrich
2,4,4 Trihydroxy-benzophenone	$C_{13}H_{10}O_4$	1470-79-7	230.22	≥95%	Sigma Aldrich
**Benzophenone-6** (2,2'-Dihydroxy-4,4'-dimethoxybenzophenone)	$C_{15}H_{14}O_5$	131-54-4	274.27	≥98%	TRC
**Dioxybenzone or Benzophenone-8** (2,2'-Dihydroxy-4-methoxybenzophenone)	$C_{14}H_{12}O_4$	131-53-3	244.24	≥98%	Sigma Aldrich
**Benzophenone-1** (2,4-Dihydroxy-benzophenone)	$C_{13}H_{10}O_3$	131-56-6	214.22	≥99%	Sigma Aldrich
4-Hydroxybenzophenone	$C_{13}H_{10}O_2$	1137-42-4	198.22	≥98%	Sigma Aldrich

Table S2. 11 isotopic labelled standards used in external standard calibration, processed blanks and follicular fluid samples.

Isotopically labelled standard	Molecular formula	CAS	MW (g/mol)	Labelled Purity	Supplier
Monoisopropyl phthalate-d4	$C_{11}H_8D_4O_4$	2733160-00-2	212.24	≥99%	CDN Isotopes
Mono-n-butyl phthalate ($^{13}C_4$, CLM4590)	$^{12}C_8{}^{13}C_4H_{14}O_4$	2687959-76-6	226.21	≥99%	Cambridge Isotope
Monocyclohexyl phthalate ($^{13}C_4$, CLM4592)	$^{12}C_{10}{}^{13}C_4H_{16}O_4$	2687959-81-3	252.25	≥95%	Cambridge Isotope
n-Propyl 4-Hydroxybenzoate-2,3,5,6-d4 (D-7114)	$C_{10}H_8D_4O_3$	1219802-67-1	184.23	≥98%	CDN Isotopes
Bisphenol S-2,2',3,3',5,5',6,6'-d8	$C_{12}H_2D_8O_4S$	2483831-28-1	258.32	≥98%	CDN Isotopes
3,3',5,5'-Tetrabromobisphenol A-d6 (dimethyl-d6)	$C_{15}H_6D_6Br_4O_2$	-	549.91	≥99%	CDN Isotopes
Triclosan-d3 (2,4-dichlorophenoxy-d3)	$C_{12}H_4D_3Cl_3O_2$	1020719-98-5	292.56	≥97%	CDN Isotopes
Triclocarban-d4 (4-chlorophenyl-d4)	$C_{13}H_9Cl_3N_2O$	1219799-29-7	319.61	≥99%	CDN Isotopes
2,4-Dihydroxybenzophenone-2',3',4',5',6'-d5	$C_{13}H_5D_5O_3$	91586-06-0	219.25	≥98%	CDN Isotopes
4-Hydroxybenzoic acid-d4	$C_7H_2D_4O_3$	152404-47-2	142.15	>95%	TRC
3,4-Dihydroxybenzoic acid methyl ester-d3 (methyl-d3)	$C_8H_5D_3O_4$	2734001-51-3	171.17	>95%	TRC

Table S3. 16 additional standards used in retention time model development, mass spectral database and/or structural confirmation in NTA.

**Analyte**	**Molecular formula**	**CAS**	**MW (g/mol)**	**Purity/ Grade**	**Supplier**
Triclosan glucuronide (sodium salt)	$C_{18}H_{14}Cl_3NaO_8$	-	487.65	>95%	TRC
Triclosan sulfate (sodium salt)	$C_{12}H_6Cl_3NaO_5S$	68508-18-9	391.59	95%	TRC
Bisphenol A glucuronide	$C_{21}H_{24}O_8$	267244-08-6	404.41	>95%	TRC
Bisphenol A sulfate (sodium salt)	$C_{15}H_{15}NaO_5S$	847696-37-1	330.33	>95%	TRC
Bisphenol B glucuronide	$C_{22}H_{26}O_8$	-	418.44	>95%	TRC
Propyl paraben sulfate (sodium salt)	$C_{10}H_{11}NaO_6S$	-	282.25	>95%	TRC
Bisphenol F sulfate (sodium salt)	$C_{13}H_{11}NaO_5S$	54172-90-6	302.28	93%	TRC
Mono-2-ethyl-5-hydroxyhexyl phthalate	$C_{16}H_{22}O_5$	40321-99-1	294.34	>90%	TRC
24 Bisphenol S	$C_{12}H_{10}O_4S$	5397-34-2	250.27	>95%	TRC
Bisphenol F	$C_{13}H_{12}O_2$	620-92-8	200.23	≥99%	Sigma Aldrich
2-Acetylbenzoic acid	$C_9H_8O_3$	577-56-0	164.16	≥99%	Sigma Aldrich
Hippuric Acid	$C_9H_9NO_3$	495-69-2	179.17	>95%	TRC
4-Hydroxyhippuric acid	$C_9H_9NO_4$	2482-25-9	195.17	>95%	TRC
Salicylic Acid	$C_7H_6O_3$	69-72-7	138.12	≥99%	AccuStandard
Phthalic acid	$C_8H_6O_4$	88-99-3	166.13	≥99%	Sigma Aldrich
1-Naphthoic acid	$C_{11}H_8O_2$	86-55-5	172.18	≥97%	Fluka Analytical

2. Methods

Treatment of 500 µL pooled follicular fluid samples. 500 µL pooled follicular fluid samples were analyzed in their unhydrolyzed and hydrolyzed forms. While the procedure was identical to the steps outlined for 100 µL follicular fluid samples, some changes were made in the amounts of chemicals and reagents to accommodate the larger sample size. Briefly, in two 15 mL centrifuge tubes, 5 µL 200 ng/mL isotopically-labelled EDC standard solution was added to each tube containing 500 µL pooled follicular fluid. A 250 µL ammonium acetate solution (1M, pH = 5.0) without enzymes was added to the unhydrolyzed sample, while another 250 µL ammonium acetate solution (1M, pH = 5.0) containing 1400 U β-glucuronidase and 140 U sulfatase was added to the hydrolyzed sample for 2-hr incubation at 37 °C. After increasing the temperature to 60 °C for 15 minutes, the hydrolyzed sample was cooled to room temperature. Then, 100 µL HPLC-grade acetic acid was added to both samples, followed by an addition of 3400 µL cold acetonitrile (at -20 ^{o}C) for protein precipitation. Both samples were stored in a -20 °C freezer for at least 60 minutes, and were centrifuged at 5000 rpm for 15 minutes. The supernatant was then filtered through Captiva EMR-Lipid cartridges (300 mg/3 mL) preconditioned with 2 mL 80% acetonitrile in water (0.1% acetic acid), and 1 mL of the same solvent was used to wash the protein pellet for a second filtration. The collected extracts were evaporated to dryness by a gentle flow of nitrogen gas, followed by 200 µL solvent reconstitution containing 50% methanol in water with 0.1% acetic acid. Samples were then filtered (0.22 µM) and centrifuged at 10000 rpm for 2 minutes prior to LC MS analysis. 500 µL HPLC-grade water with and without enzymatic hydrolysis were used as process blanks. The entire procedure was repeated for duplicate analysis to ensure consistency.

Retention time prediction model. Retention time prediction was performed through Quantitative Structure-Retention Relationship (QSRR) modeling. To do this, all 80 standards listed in **Table S1** and **Table S3** were converted to more than 5000 molecular descriptors calculated through alvaDesc software. These descriptors reflect the physiochemical properties that affect the retention time of the analytes.[1] The training set and testing test consisted of 66 and 14 different compounds, respectively. Compared to our previous protocol,[1] we made the following adjustments to the descriptor elimination rule. First, a linear regression coefficient (R^2) between RT and any single descriptor could be below 0.6. Second, descriptor co-linearity was assessed, and no pair of descriptors had an R^2 value above 0.6. Molecular descriptors were selected through forward stepwise regression for establishing a multiple linear regression equation (eq. S1).[1,2]

$$\mathrm{RT}_{\text{predicted}} = C + a_1M_1 + a_2M_2 + a_3M_3 + \cdots + a_nM_n \quad \text{(eq. S1)}$$

where *C* is the constant; a_1, a_2, …, a_n are coefficients, and M_1, M_2, …, M_n are values of the molecular descriptors. *C* and coefficients were calculated through the LINEST function in Excel (**Table S4**).

Table S4. Constant, coefficients, and molecular descriptors used in the retention time model equation.

Constant/Molecular descriptor symbol	Value/coefficient
Constant (*C*)	-39.3001
ALOGP	0.8741
MATS1v	7.3025
MATS4e	5.4987
GATS2e	2.6839
Eta_D_epsiD	-121.8183
ATSC2e	4.6798
SpMAD_AEA(bo)	-9.7071
MATS1m	-8.7624
GATS5v	-0.9112
JGT	7.6616
VR2_Dz(p)	45.2524
GATS6s	0.5446
P_VSA_MR_2	0.0197
SpMax4_Bh(s)	-0.7895
PW4	-4.4613
IC4	3.4346
AAC	7.3464
SpMax8_Bh(i)	2.2889
SM11_AEA(bo)	-3.3545
MATS3p	-3.0195
GATS1s	3.9041
rGes	-5.2886
GGI4	-3.2321
MATS4p	2.3108
J_Dz(p)	1.3935
VE1_D/Dt	0.5029
Eta_sh_y	8.1006
C%	-0.1172

In terms of model performance (**Figure S1** and **Table S5**), R^2 was >0.99 between the experimental and model predicted RTs for 66 chemicals in the training set. For the 14 compounds in the testing set, 50% of compounds (n = 7) had their predicted RTs within 0.5 min of the actual experimental values. This matching rate increased to 79% (n = 11) when the threshold was set to 1.0 min. It should be noted that the present model is only restricted to the LC-MS conditions reported in this work.

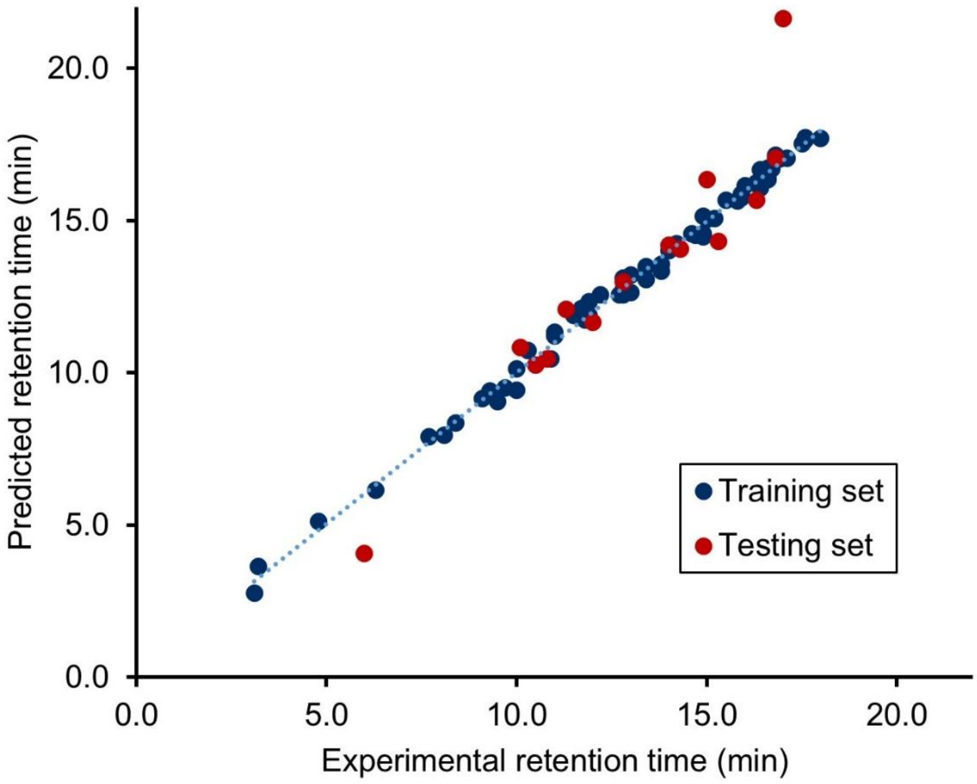

Figure S1. Comparison of experimental and predicted retention time of chemicals in the trainings set and testing set.

Table S5. Detailed comparison of experimental and predicted retention time of chemicals in the testing set.

**Chemical**	**Measured retention time (min)**	**Predicted retention time (min)**	**Difference (ΔRT, min)**
Monoethyl phthalate	10.5	10.3	0.2
1-Naphthoic acid	12.8	13.0	0.2
Mono-2-ethyl-5-hydroxyhexyl phthalate	14.0	14.2	0.2
Benzophenone-1	14.3	14.1	0.2
Ethyl paraben	10.8	10.5	0.3
Propyl paraben sulfate	12.0	11.7	0.3
Monooctyl phthalate	16.8	17.1	0.3
Bisphenol G	16.3	15.7	0.6
24 Bisphenol S	10.1	10.8	0.7
Bisphenol F	11.3	12.1	0.8
2,4,6-Tribromophenol	15.3	14.3	1.0
Mono(carboxyisooctyl) phthalate	15.0	16.3	1.3
Phthalic acid	6.0	4.1	1.9
Bithionol	17.0	21.6	4.6

Recovery and matrix effect calculations. All quantitation was performed on the Tracefinder software (Thermo Fisher Scientific). The calibration curve of each standard was established through eq. S2.[3]

$$\frac{C_{\text{analyte}}}{C_{\text{ISTD}}} = k \times \frac{A_{\text{analyte}}}{A_{\text{ISTD}}} \quad \text{(eq. S2)}$$

where C_{analyte} is the concentration of the standard; C_{ISTD} is the concentration of internal standard; k is the slope of the calibration curve; A_{analyte} and A_{ISTD} are the precursor ion peak areas of the analyte and internal standard, respectively. Since not every analyte had its corresponding isotopically labelled standard available, an internal standard with the highest degree of structural similarity was chosen. For example, n-propyl paraben-d4 was used as the internal standard for all 10 parabens. Note that the number of internal standards used in this work was purposely limited in order to avoid analyte ion suppression in the C-trap. With the use of slope k obtained in the calibration curves, the concentrations of each analyte ($C_{\text{analyte-sample}}$) in follicular fluid samples were obtained through eq. S3.[3]

$$\frac{C_{\text{analyte-sample}}}{C_{\text{ISTD-sample}}} = k \times \frac{A_{\text{analyte-sample}}}{A_{\text{ISTD-sample}}} \quad \text{(eq. S3)}$$

To evaluate procedural losses, the recovery (R) of each analyte in the spiked samples was calculated using eq. S4.[4] Note that this parameter is also known as accuracy or global matrix effect by some studies.[4]

$$R = \frac{C_{(0+S)} - C_0}{C_S} \times 100\% \quad \text{(eq. S4)}$$

where $C_{(0+S)}$ is the calculated concentration of each analyte in the spiked samples; C_0 is the calculated concentration of each analyte in unspiked samples; C_S is the spiking concentration (1 ng, 5 ng or 25 ng/mL). Lastly, from eq. S5,[5,6] the impacts of chemical extraction and clean-up processes were evaluated through instrumental matrix effect (ME), which compares analyte signals in post-extraction spiked follicular fluid samples with those in neat standard solutions. Specifically, 6 blank matrix samples (100 µL follicular fluid without any spiked standards) were treated. The EDC standards (0, 0.5, 1, 5, 10, 25 ng/mL) and internal standards (5 ng/mL in all samples) were added during reconstitution in 200 µL of 50% methanol in water containing 0.1% acetic acid. A minimum of three datapoints were used for ME calculations.

$$ME = \left(\frac{A_{\text{post-spike}} - A_{\text{blank matrix}}}{A_{\text{solvent}}}\right) \times 100\% \quad \text{(eq. S5)}$$

where $A_{\text{post-spike}}$ is the peak area of analytes in post-spiked follicular fluid samples (> 0 ng/mL); $A_{blank\ matrix}$ is analyte peak area in blank matrix (the original analytes in follicular fluid without any spiking), and A_{solvent} is the analyte peak area in neat solvent, respectively. An ME greater than 1 corresponds to signal enhancement

whereas an ME smaller than 1 represents signal suppression. The ME is considered as insignificant when the calculated ME is between 80% and 120%.[5]

Key Compound Discoverer settings.

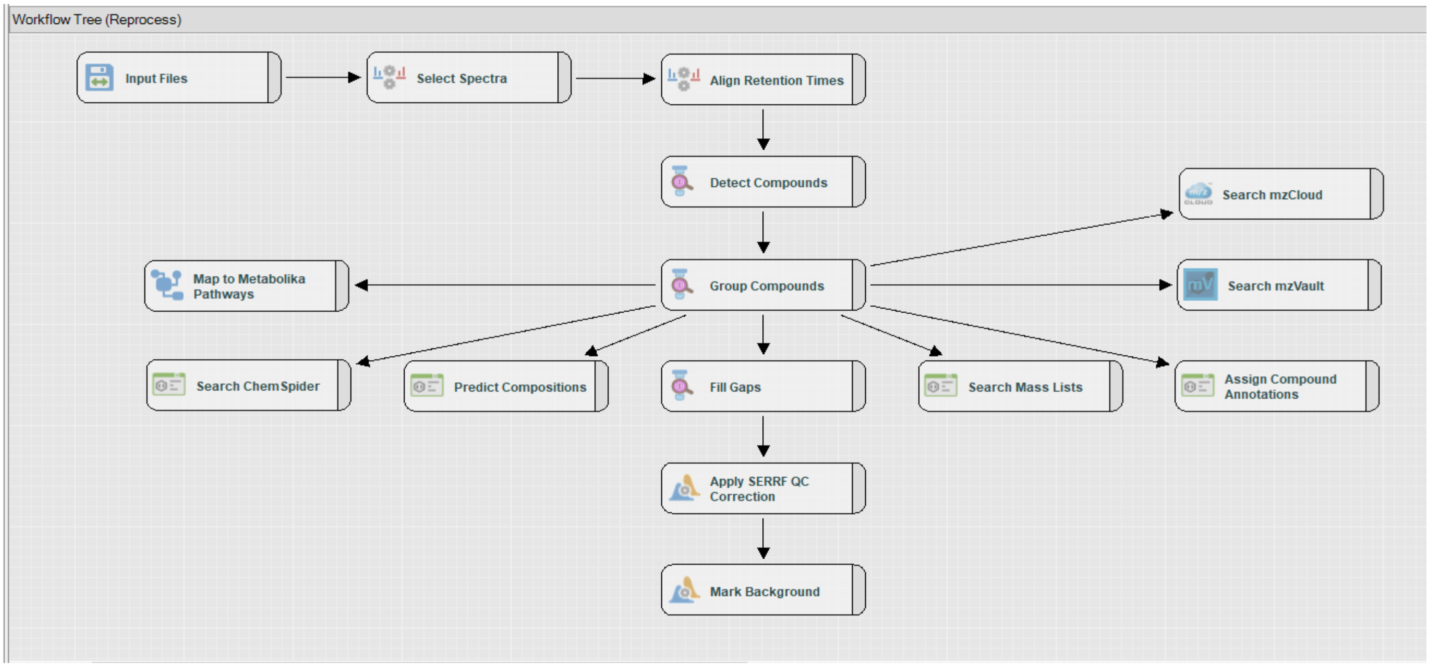

Figure S2. Compound Discoverer workflow tree in this study. Detailed settings in each node are listed below.

Processing node 6: Input Files

Input Data:

- File Name(s) (Hidden):

Processing node 33: Select Spectra

----

1. Spectrum Properties Filter:

- Lower RT Limit: 2.5

- Upper RT Limit: 20

- First Scan: 0

- Last Scan: 0

- Ignore Specified Scans: (not Specified)
- Total Intensity Threshold: 0
- Minimum Peak Count: 1

1.1 Spectrum Properties Filter for DDA Spectra:
- Lowest Charge State: 0
- Highest Charge State: 0
- Min. Precursor Mass: 100 Da
- Max. Precursor Mass: 800 Da

2. Scan Event Filters:
- Mass Analyzer: (not Specified)
- MS Order: Any
- Activation Type: (not Specified)
- Acquisition Type: (not Specified)
- Min. Collision Energy: 0
- Max. Collision Energy: 1000
- Scan Type: Any
- Polarity Mode: (not Specified)
- MS1 Mass Range: (not Specified)
- FAIMS CV: (not Specified)

3. Peak Filters:
- S/N Threshold (FT-only): 3

4. Replacements for Unrecognized Properties:
- Unrecognized Charge Replacements: 1
- Unrecognized Mass Analyzer Replacements: ITMS
- Unrecognized MS Order Replacements: MS2
- Unrecognized Activation Type Replacements: HCD
- Unrecognized Polarity Replacements: -
- Unrecognized MS Resolution@200 Replacements: 60000

- Unrecognized MSn Resolution@200 Replacements: 30000

6. General Settings:

- Precursor Selection: Use MS(@N - 1) Precursor
- Use Isotope Pattern in Precursor Reevaluation: True
- Provide Profile Spectra: Automatic
- Spectra to Store: all
- Store Chromatograms: False

Processing node 48: Align Retention Times

----

1. General Settings:

- Alignment Model: Adaptive curve
- Alignment Fallback: None
- Maximum Shift [min]: 0.3
- Shift Reference File: True
- Mass Tolerance: 5 ppm
- Remove Outlier: False

Processing node 47: Detect Compounds

----

1. General Settings:

- Mass Tolerance [ppm]: 5 ppm
- Min. Peak Intensity: 10000
- Min. # Scans per Peak: 3
- Use Most Intense Isotope Only: True
- Precursor Mass Tolerance: 0.025 Da

2. Trace Detection:

- Max. Number of Gaps to Correct: 2

- Min. Number of Adjacent Non-Zeros: 2
- Trace Mass Update Strategy: Weighted Mean

3. Peak Detection:
- Chromatographic S/N Threshold: 3
- Remove Baseline: False
- Gap Ratio Threshold: 0.35
- Max. Peak Width [min]: 1
- Min. Relative Valley Depth: 0.1

4. Isotope Pattern Detection:
- Group Isotopes for: Br; Cl
- RT Tolerance [min]: 0
- Use Peak Quality for Isotope Grouping: True
- Filter out Features with Bad Peaks Only: True
- Zig-Zag Index Threshold: 0.2
- Jaggedness Threshold: 0.4
- Modality Threshold: 0.9
- Remove Potentially False Positive Isotopes: True

5. Compound Assembly:
- Ions: [M-H]-1
- Base Ions: [M-H]-1
- Remove Singlets: True

6. AcquireX Settings:
- Detect Persistent Background Ions: False

Processing node 31: Group Compounds

----

1. General Settings:

- Mass Tolerance: 5 ppm
- RT Tolerance [min]: 0.3
- Minimum Valley [%]: 10
- Align Peaks: False
- Preferred Ions: [M-H]-1
- Area Integration: Most Common Ion

2. Peak Rating Contributions:
- Area Contribution: 3
- CV Contribution: 10
- FWHM to Base Contribution: 5
- Jaggedness Contribution: 5
- Modality Contribution: 5
- Zig-Zag Index Contribution: 5

3. Peak Rating Filter:
- Peak Rating Threshold: 4
- Number of Files: 1

Processing node 32: Fill Gaps

----

1. General Settings:
- Mass Tolerance: 5 ppm
- S/N Threshold: 3
- Use Real Peak Detection: True
- Apply Restrictive Gap Filling: True
- Min. # Scans per Peak: 3

Processing node 45: Apply SERRF QC Correction

This node was not relevant to this study. Default values were kept in the workflow.

Processing node 28: Mark Background

----

1. General Settings:

- Max. Sample/Blank: 3
- Max. Blank/Sample: 0
- Hide Background: True

Processing node 25: Assign Compound Annotations

----

1. General Settings:

- Mass Tolerance: 5 ppm

2. Data Sources:

- Data Source #1: mzVault Search
- Data Source #2: mzCloud Search
- Data Source #3: Predicted Compositions
- Data Source #4: MassList Search
- Data Source #5: ChemSpider Search
- Data Source #6: (not specified)
- Data Source #7: (not specified)

3. Scoring Rules:

- Use mzLogic: True
- Use Spectral Distance: True
- SFit Threshold: 50
- SFit Range: 50

4. Reprocessing:

- Clear Names: False

Processing node 42: Search mzCloud

----

1. General Settings:

- Compound Classes:

    Endogenous Metabolites

    Excipients/Additives/Colorants

    Industrial Chemicals

    Natural Products/Medicines

    Natural Toxins

    Personal Care Products/Cosmetics

    Pesticides/Herbicides

    Small Molecule Chemicals

    Steroids/Vitamins/Hormones

    Textile Chemicals/Auxiliary/Dyes

    Therapeutics/Prescription Drugs

- Precursor Mass Tolerance: 5 ppm
- FT Fragment Mass Tolerance: 10 ppm
- IT Fragment Mass Tolerance: 0.4 Da
- Library: Autoprocessed; Reference
- Post Processing: Recalibrated
- Max. # Results: 10
- Annotate Matching Fragments: True
- Search MSn Tree: True

2. DDA Search:

- Identity Search: HighChem HighRes
- Match Activation Type: True
- Match Activation Energy: Any
- Activation Energy Tolerance: 20

- Apply Intensity Threshold: True
- Similarity Search: Confidence Forward
- Match Factor Threshold: 50

3. DIA Search:
- Use DIA Scans for Search: False
- Max. Isolation Width [Da]: 500
- Match Activation Type: False
- Match Activation Energy: Any
- Activation Energy Tolerance: 100
- Apply Intensity Threshold: False
- Match Factor Threshold: 20

Processing node 41: Search ChemSpider

This node was not relevant to this study. Default values were kept in the workflow.

Processing node 43: Map to Metabolika Pathways

This node was not relevant to this study. Default values were kept in the workflow.

Processing node 49: Search Mass Lists

----

1. Search Settings:
- Mass Lists:
    HMDB All metabolites (v5. 217719 cpds).massList
    HMDB Serum metabolites (v5. 40600 cpds).massList
    HMDB Urine metabolites (v5. 4641 cpds).massList
- Use Retention Time: True

- RT Tolerance [min]: 2
- Mass Tolerance: 5 ppm

Processing node 50: Search mzVault

----

1. Search Settings:
- mzVault Library: *in-house database, file name hidden*
- Max. # Results: 10
- Match Factor Threshold: 50
- Search Algorithm: HighChem HighRes
- Match Analyzer Type: True
- IT Fragment Mass Tolerance: 0.4 Da
- FT Fragment Mass Tolerance: 10 ppm
- Use Retention Time: False
- Precursor Mass Tolerance: 5 ppm
- Apply Intensity Threshold: True
- Match Ionization Method: True
- Ion Activation Energy Tolerance: 20
- Match Ion Activation Energy: Match with Tolerance
- Match Ion Activation Type: True
- Compound Classes: all
- Remove Precursor Ion: True
- RT Tolerance [min]: 2

Processing node 40: Predict Compositions

----

1. Prediction Settings:
- Mass Tolerance: 5 ppm
- Min. Element Counts: C H O
- Max. Element Counts: C90 H190 BR3 CL4 N10 O18 P3 S5

- Min. RDBE: 0
- Max. RDBE: 40
- Min. H/C: 0.1
- Max. H/C: 3.5
- Max. # Candidates: 10
- Max. # Internal Candidates: 200

2. Pattern Matching:
- Intensity Tolerance [%]: 30
- Intensity Threshold [%]: 0.1
- S/N Threshold: 3
- Min. Spectral Fit [%]: 30
- Min. Pattern Cov. [%]: 90
- Use Dynamic Recalibration: True

3. Fragments Matching:
- Use Fragments Matching: True
- Mass Tolerance: 5 ppm
- S/N Threshold: 3

Processing node 17: Differential Analysis

This node was not relevant to this study. Default values were kept in the workflow.

3. Diagnostic fragmentation of paraben ions

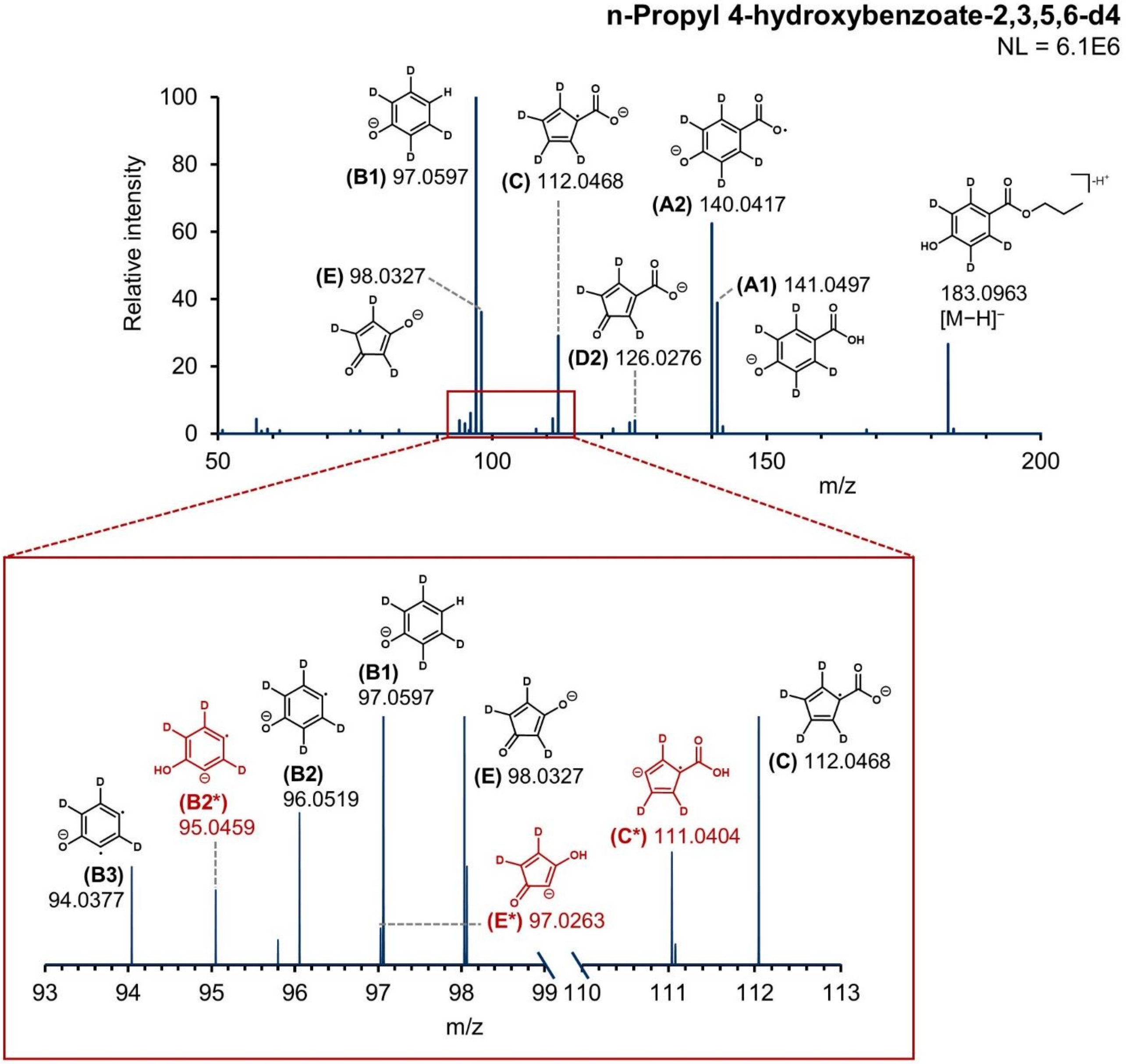

Figure S3. MS/MS spectrum of 50 ng/mL n-propyl 4-hydroxybenzoate-2,3,5,6-d4 at 20 eV collision energy. The ion labels are identical to the ones used for non-deuterated fragmentation ions shown in Figure 4. Product ions (with *) highlighted in red represent the less abundant ions with deuterium loss.

Table S6. Precursor ions and major product ions of parabens and their phase I metabolites at different collision energies in MS/MS. All m/z shown are the accurate monoisotopic masses.

Standard name	Molecular formula	Precursor ion [M–H]⁻	RT (min)	Major product ions		
				10 eV CE	20 eV CE	40 eV CE
**MeP** (Methyl paraben)	$C_8H_8O_3$	151.0395	9.4	95.0140<br>108.0217<br>136.0166	91.0190<br>92.0269<br>95.0140<br>108.0218<br>123.0090<br>136.0166	91.0188<br>92.0272<br>95.0140<br>108.0218<br>123.0094
**EtP** (Ethyl paraben)	$C_9H_{10}O_3$	165.0552	10.8	93.0346<br>136.0166<br>137.0245	91.0190<br>92.0270<br>93.0347<br>95.0140<br>108.0218<br>136.0166<br>137.0245	91.0192<br>92.0269<br>93.0346<br>95.0140<br>108.0217<br>123.0091
**iPrP** (Isopropyl paraben)	$C_{10}H_{12}O_3$	179.0708	11.8	93.0347<br>136.0167<br>137.0245	92.0272<br>93.0347<br>95.0140<br>108.0219<br>136.0168<br>137.0246	93.0347<br>95.0140<br>108.0218<br>123.0087
**PrP** (Propyl paraben)	$C_{10}H_{12}O_3$	179.0708	12.2	93.0346<br>136.0166<br>137.0245	91.0190<br>92.0270<br>93.0347<br>95.0140<br>108.0218<br>123.0086<br>136.0166<br>137.0245	91.0189<br>92.0269<br>93.0347<br>95.0140<br>108.0218<br>123.0090
**iBuP** (Isobutyl paraben)	$C_{11}H_{14}O_3$	193.0865	13.4	136.0167	91.0191<br>92.0269<br>93.0347<br>108.0219<br>123.0090<br>136.0167<br>137.0246	91.0190<br>92.0270<br>93.0347<br>95.0140<br>108.0219<br>123.0091
**BuP** (Butyl paraben)	$C_{11}H_{14}O_3$	193.0865	13.7	93.0346<br>136.0166<br>137.0244	91.0191<br>92.0269<br>93.0347<br>95.0140<br>108.0218<br>136.0166<br>137.0245	91.0190<br>93.0347<br>95.0140<br>108.0218<br>123.0089
**iPeP** (Isopentyl paraben)	$C_{12}H_{16}O_3$	207.1021	14.9	93.0347<br>136.0167	91.0191<br>92.0272	93.0347<br>95.0140

				137.0245	93.0347<br>95.0140<br>108.0219<br>136.0168<br>137.0246	108.0218<br>123.0087
**BzP** (Benzyl paraben)	$C_{14}H_{12}O_3$	227.0708	14.8	136.0166	92.0269<br>95.0140<br>108.0218<br>123.0091<br>136.0166	92.0271<br>95.0140<br>108.0219<br>123.0093
**2-EtHeP** (2-ethylhexyl paraben)	$C_{15}H_{22}O_3$	249.1491	16.6	136.0166	91.0191<br>92.0269<br>93.0347<br>95.0140<br>108.0218<br>136.0167<br>137.0245	91.0191<br>92.0269<br>93.0347<br>95.0140<br>108.0218<br>123.0089<br>136.0167
**OcP** (n-Octyl paraben)	$C_{15}H_{22}O_3$	249.1491	16.8	137.0246	91.0189<br>92.0268<br>93.0346<br>95.0139<br>108.0217<br>136.0165<br>137.0244	91.0191<br>92.0268<br>93.0346<br>95.0139<br>108.0217
**4-HB** (4-Hydroxy benzoic acid)	$C_7H_6O_3$	137.0239	4.7	93.0347	93.0347	93.0347
**3,4-DHB** (3,4-Dihydroxybenzoic acid)	$C_7H_6O_4$	153.0188	3.1	109.0294	91.0189<br>108.0217<br>109.0294	91.0191<br>108.0217<br>109.0298
**OH-MeP** (Methyl 3,4-dihydroxybenzoate or Methyl protocatechuate)	$C_8H_8O_4$	167.0344	7.7	152.0115	83.0139<br>95.0139<br>107.0138<br>108.0218<br>111.0087<br>123.0088<br>124.0166<br>139.0036<br>152.0114	83.0138<br>91.0190<br>95.0139<br>107.0138<br>111.0086<br>123.0089<br>124.0166<br>139.0033
**OH-EtP** (Ethyl 3,4-dihydroxybenzoate or Ethyl protocatechuate)	$C_9H_{10}O_4$	181.0501	9.5	109.0294<br>152.0115<br>153.0193	83.0140<br>95.0139<br>108.0217<br>109.0294<br>111.0087<br>123.0087<br>124.0165<br>139.0037<br>152.0115<br>153.0193	83.0138<br>91.0190<br>95.0139<br>107.0139<br>108.0217<br>109.0295<br>111.0088<br>123.0087<br>124.0165<br>139.0034

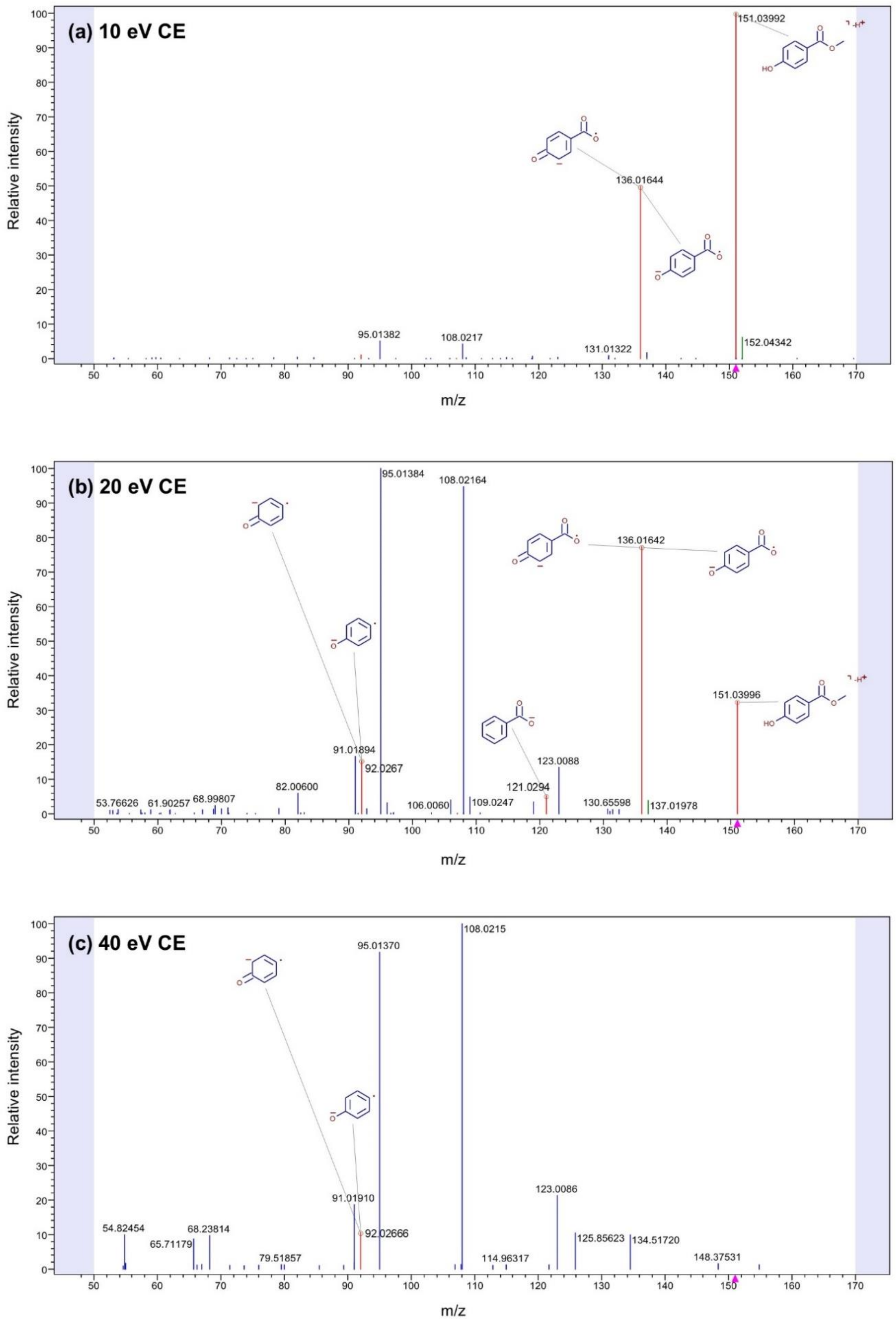

Figure S4. Experimental tandem MS/MS spectra of 50 ng/mL methyl paraben at **(a)** 10 eV, **(b)** 20 eV and **(c)** 40 eV collision energy. The peaks highlighted in red represent matching precursor and fragmentation ions identified in both experimental and predicted spectra using the Mass Frontier 8.0 software (Thermo Fisher Scientific).

4. Compound identification in spiked follicular fluid

Table S7. Mass-to-charge ratios, measured retention times, estimated instrument detection limit (IDL, ng per mL solvent) and method detection limit (MDL, ng per mL follicular fluid) of EDC standards spiked in follicular fluid. IDL was estimated from 8 standard solutions with concentrations ranging from 0.2 ng/mL to 50 ng/mL, and MDL was estimated from 3 levels of spiked follicular fluid samples (1, 5 and 25 ng/mL follicular fluid). ND = Not detected.

Standard name	Molecular formula	Precursor ion [M–H]⁻	Retention time (min)	IDL (ng/mL solvent)	MDL (ng/mL follicular fluid)
**MHBP** [Mono (3-hydroxybutyl) phthalate]	$C_{12}H_{14}O_5$	237.0763	10.0	0.2	1
Mono (3-carboxypropyl) phthalate	$C_{12}H_{12}O_6$	251.0556	10.0	2	25
**MMP** (Monomethyl phthalate)	$C_9H_8O_4$	179.0344	9.1	0.5	5
Monomethyl isophthalate	$C_9H_8O_4$	179.0344	11.0	0.2	5
**MEP** (Monoethyl phthalate)	$C_{10}H_{10}O_4$	193.0501	10.5	0.2	5
Monoisopropyl phthalate	$C_{11}H_{12}O_4$	207.0657	11.4	0.2	1
**MBP** (Monobutyl phthalate)	$C_{12}H_{14}O_4$	221.0814	13.4	5	25
**MIBP** (Monoisobutyl phthalate)	$C_{12}H_{14}O_4$	221.0814	13.0	0.5	5
Mono(carboxyisooctyl) phthalate	$C_{17}H_{22}O_6$	321.1338	15.0	0.5	1
Mono-2-carboxy-methylhexyl phthalate	$C_{16}H_{20}O_6$	307.1182	14.5	1	25
**MBzP** (Monobenzyl phthalate)	$C_{15}H_{12}O_4$	255.0657	14.7	0.2	1
**MCHP** (Monocyclohexyl phthalate)	$C_{14}H_{16}O_4$	247.0970	15.0	0.2	1
Mono-n-pentyl phthalate	$C_{13}H_{16}O_4$	235.0970	14.9	0.2	1
Mono-9-carboxynonyl phthalate	$C_{18}H_{24}O_6$	335.1495	15.9	0.2	5
Monohexyl phthalate	$C_{14}H_{18}O_4$	249.1127	15.8	0.2	5
Monoheptyl phthalate	$C_{15}H_{20}O_4$	263.1283	16.4	0.2	1
Monooctyl phthalate	$C_{16}H_{22}O_4$	277.1440	16.8	0.2	5
Mono-5-carboxypentyl phthalate	$C_{14}H_{16}O_6$	279.0869	11.9	0.5	5
Monopropyl phthalate	$C_{11}H_{12}O_4$	207.0657	11.8	0.2	1
**MEHP** [Mono (2-ethylhexyl) phthalate]	$C_{16}H_{22}O_4$	277.1440	16.6	5	5
**MeP** (Methyl paraben)	$C_8H_8O_3$	151.0395	9.4	0.2	1
**EtP** (Ethyl paraben)	$C_9H_{10}O_3$	165.0552	10.8	0.2	1
**iPrP** (Isopropyl paraben)	$C_{10}H_{12}O_3$	179.0708	11.8	0.5	1
**PrP** (Propyl paraben)	$C_{10}H_{12}O_3$	179.0708	12.2	0.2	1

**iBuP** (Isobutyl paraben)	$C_{11}H_{14}O_3$	193.0865	13.4	0.2	1
**BuP** (Butyl paraben)	$C_{11}H_{14}O_3$	193.0865	13.7	0.2	1
**iPeP** (Isopentyl paraben)	$C_{12}H_{16}O_3$	207.1021	14.9	0.2	5
**BzP** (Benzyl paraben)	$C_{14}H_{12}O_3$	227.0708	14.9	0.2	5
**2-EtHeP** (2-ethylhexyl paraben)	$C_{15}H_{22}O_3$	249.1491	16.6	0.2	5
**OcP** (n-Octyl paraben)	$C_{15}H_{22}O_3$	249.1491	16.8	0.2	1
**4-HB** (4-Hydroxy benzoic acid)	$C_7H_6O_3$	137.0239	4.8	0.2	5
**3,4-DHB** (3,4-Dihydroxybenzoic acid)	$C_7H_6O_4$	153.0188	3.1	1	5
**OH-MeP** (Methyl 3,4-dihydroxybenzoate or Methyl protocatechuate)	$C_8H_8O_4$	167.0344	7.7	0.2	1
**OH-EtP** (Ethyl 3,4-dihydroxybenzoate or Ethyl protocatechuate)	$C_9H_{10}O_4$	181.0501	9.5	0.2	1
**BPA** (Bisphenol A)	$C_{15}H_{16}O_2$	227.1072	12.8	ND	ND
**BPB** (Bisphenol B)	$C_{16}H_{18}O_2$	241.1229	14.2	ND	ND
**BPS** (Bisphenol S)	$C_{12}H_{10}O_4S$	249.0222	9.3	0.2	1
**TDP** (4,4'-Thiodiphenol)	$C_{12}H_{10}O_2S$	217.0323	11.9	2	5
Bisphenol TMC	$C_{21}H_{26}O_2$	309.1855	16.6	10	>25
**BHPF** (Bisphenol FL)	$C_{25}H_{18}O_2$	349.1229	16.5	5	>25
**BPG** (Bisphenol G)	$C_{21}H_{28}O_2$	311.2011	16.3	10	>25
Bisphenol BP	$C_{25}H_{20}O_2$	351.1385	16.7	2	25
Bisphenol PH	$C_{27}H_{24}O_2$	379.1698	17.6	5	>25
**BPM** (Bisphenol M)	$C_{24}H_{26}O_2$	345.1855	16.9	0.2	5
**BPP** (Bisphenol P)	$C_{24}H_{26}O_2$	345.1855	17.1	0.5	5
4-Phenylphenol	$C_{12}H_{10}O$	169.0653	14.0	5	>25
Dichlorophen	$C_{13}H_{10}Cl_2O_2$	266.9980	15.2	0.2	1
Hexachlorophen	$C_{13}H_6Cl_6O_2$	404.8391	17.6	0.2	5
Bithionol	$C_{12}H_6Cl_4O_2S$	354.8735	17.0	0.5	5
**TBBPA** (3,3',5,5',-Tetrabromobisphenol A)	$C_{15}H_{12}Br_4O_2$	542.7452	17.4	0.2	25
**TBBPS** (Tetrabromobisphenol S)	$C_{12}H_6Br_4O_4S$	564.6615	15.9	0.2	>25
Triclosan	$C_{12}H_7Cl_3O_2$	286.9433	16.8	0.2	1
2,4-Dichlorophenol	$C_6H_4Cl_2O$	160.9561	11.7	1	>25
2,4,6-Trichlorophenol	$C_6H_3Cl_3O$	194.9171	13.7	1	>25
2,4-Dibromophenol	$C_6H_4Br_2O$	250.8530	12.7	0.5	25
2,4,6-Tribromophenol	$C_6H_3Br_3O$	328.7635	15.3	0.5	5
4,6-Dichlororesorcinol	$C_6H_4Cl_2O_2$	176.9510	9.6	0.2	1
Triclocarban	$C_{13}H_9Cl_3N_2O$	312.9702	16.3	0.5	5
**Benzophenone-2** (2,2,4,4 Tetrayhydroxy-benzophenone)	$C_{13}H_{10}O_5$	245.0450	10.3	0.2	1

2,4,4 Trihydroxy-benzophenone	$C_{13}H_{10}O_4$	229.0501	10.9	0.2	1
**Benzophenone-6** (2,2'-Dihydroxy-4,4'-di-methoxybenzophenone)	$C_{15}H_{14}O_5$	273.0763	16.6	0.2	5
**Dioxybenzone or Benzophenone-8** (2,2'-Dihydroxy-4-methoxybenzophenone)	$C_{14}H_{12}O_4$	243.0657	15.5	0.2	5
**Benzophenone-1** (2,4-Dihydroxy-benzophenone)	$C_{13}H_{10}O_3$	213.0552	14.3	0.2	1
4-Hydroxybenzophenone	$C_{13}H_{10}O_2$	197.0603	12.8	0.2	1

Table S8. Mass-to-charge ratios, measured retention times and relative standard deviation (RSD, %) of internal standards signal response in calibration standards (n = 8) and follicular fluid samples (n = 12).

**Internal standard**	**Molecular formula**	**Precursor ion [M–H]⁻**	**Retention time (min)**	**RSD in calibration standards (%)**	**RSD in follicular fluid (%)**
**PrP-d4** (n-Propyl 4-Hydroxybenzoate-2,3,5,6-d4)	$C_{10}H_8D_4O_3$	183.0959	12.1	4	15
**4-HB-d4** (4-Hydroxybenzoic acid-d4)	$C_7H_2D_4O_3$	141.0490	4.7	4	20
**OH-MeP-d3** [3,4-Dihydroxybenzoic acid methyl ester-d3 (methyl-d3)]	$C_8H_5D_3O_4$	170.0533	7.7	3	13
Monoisopropyl phthalate-d4	$C_{11}H_8D_4O_4$	211.0908	11.4	3	18
Mono-n-butyl phthalate ($^{13}C_4$)	$^{12}C_8{}^{13}C_4H_{14}O_4$	225.0948	13.4	3	12
Monocyclohexyl phthalate ($^{13}C_4$)	$^{12}C_{10}{}^{13}C_4H_{16}O_4$	251.1105	15.0	4	18
Bisphenol S-2,2',3,3',5,5',6,6'-d8	$C_{12}H_2D_8O_4S$	257.0724	9.2	2	17
3,3',5,5'-Tetrabromobisphenol A-d6 (dimethyl-d6)	$C_{15}H_6D_6Br_4O_2$	548.7828	17.4	3	15
Triclosan-d3 (2,4-dichlorophenoxy-d3)	$C_{12}H_4D_3Cl_3O_2$	289.9622	16.8	5	19
Triclocarban-d4 (4-chlorophenyl-d4)	$C_{13}H_5D_4Cl_3N_2O$	316.9953	16.3	7	16
2,4-Dihydroxybenzophenone-2',3',4',5',6'-d5	$C_{13}H_5D_5O_3$	218.0866	14.2	3	14

Table S9. Recoveries (%) and instrumental matrix effect (%) of EDC standards in the spiked 100 µL follicular fluid samples. Three levels of spikes (1, 5 and 25 ng/mL follicular fluid) were prepared for the pre-spiked samples, and six levels of spikes (0, 0.5, 1, 5, 10 and 25 ng/mL after solvent reconstitution) were prepared for the post-spiked samples. Numbers listed in parenthesis correspond to the RSD.

**Analyte**	**Recovery (1 ng spike/mL)**	**Recovery (5 ng spike/mL)**	**Recovery (25 ng spike/mL)**	**Matrix effect (%)**
**MHBP** [Mono (3-hydroxybutyl) phthalate]	87 (4)	97 (2)	103 (6)	71 (5)
Mono (3-carboxypropyl) phthalate	ND	69 (14)	71 (30)	57 (27)
Monomethyl phthalate	ND	116 (9)	107 (3)	75 (7)
Monomethyl isophthalate	56 (2)	80 (5)	100 (6)	69 (9)
Monoethyl phthalate	95 (2)	104 (3)	104 (3)	73 (14)
Monoisopropyl phthalate	95 (4)	104 (4)	106 (7)	80 (14)
Monobutyl phthalate	115 (2)	100 (3)	97 (4)	77 (7)
**MIBP** (Monoisobutyl phthalate)	93 (10)	103 (5)	99 (10)	78 (8)
Mono(carboxyisooctyl) phthalate	79 (7)	101 (6)	102 (10)	70 (12)
Mono-2-carboxy-methylhexyl phthalate	ND	99 (5)	110 (9)	77 (15)
Monobenzyl phthalate	64 (4)	93 (3)	104 (5)	69 (8)
**MCHP** (Monocyclohexyl phthalate)	93 (8)	102 (8)	102 (5)	73 (11)
Mono-n-pentyl phthalate	56 (10)	96 (6)	102 (6)	71 (9)
Mono-9-carboxynonyl phthalate	41 (3)	90 (6)	92 (12)	67 (13)
Monohexyl phthalate	93 (5)	93 (7)	104 (7)	71 (12)
Monoheptyl phthalate	47 (33)	93 (3)	103 (7)	71 (16)
Monooctyl phthalate	ND	92 (11)	111 (6)	75 (14)
Mono-5-carboxypentyl phthalate	ND	89 (4)	91 (18)	65 (8)
Monopropyl phthalate	101 (13)	98 (4)	107 (6)	76 (10)
**MEHP** [Mono(2-ethylhexyl) phthalate]	52 (4)	81 (7)	91 (7)	76 (15)
**MeP** (Methyl paraben)	144 (3)	233 (2)	243 (6)	95 (17)
**EtP** (Ethyl paraben)	90 (14)	114 (3)	118 (9)	84 (7)
**iPrP** (Isopropyl paraben)	70 (14)	107 (6)	125 (7)	74 (9)
**PrP** (Propyl paraben)	81 (3)	97 (3)	107 (5)	85 (4)
**iBuP** (Isobutyl paraben)	47 (13)	65 (10)	75 (7)	84 (7)
**BuP** (Butyl paraben)	21 (58)	69 (9)	79 (8)	86 (4)
**iPeP** (Isopentyl paraben)	13 (74)	57 (10)	72 (12)	99 (10)
**BzP** (Benzyl paraben)	ND	18 (5)	27 (7)	95 (10)

**2-EtHeP** (2-ethylhexyl paraben)	ND	34 (35)	66 (10)	87 (15)
**OcP** (n-Octyl paraben)	75 (19)	82 (26)	113 (6)	88 (11)
**4-HB** (4-Hydroxy benzoic acid) [Note 2]	ND	149 (7)	221 (2)	85 (15)
**3,4-DHB** (3,4-Dihydroxybenzoic acid) [Note 2]	ND	191 (8)	143 (4)	118 (18)
**OH-MeP** (Methyl 3,4-dihydroxybenzoate or Methyl protocatechuate)	105 (9)	104 (1)	98 (6)	93 (9)
**OH-EtP** (Ethyl 3,4-dihydroxybenzoate or Ethyl protocatechuate)	111 (6)	136 (10)	140 (10)	92 (10)
**BPA** (Bisphenol A)	ND	ND	ND	[Note 1]
**BPB** (Bisphenol B)	ND	ND	ND	[Note 1]
**BPS** (Bisphenol S)	81 (7)	109 (2)	111 (14)	83 (13)
**TDP** (4,4'-Thiodiphenol)	ND	81 (2)	108 (10)	73 (11)
Bisphenol TMC	ND	ND	67 (15)	[Note 1]
**BHPF** (Bisphenol FL)	ND	54 (17)	88 (4)	45 (47)
**BPG** (Bisphenol G)	ND	ND	81 (13)	[Note 1]
Bisphenol BP	ND	59 (19)	96 (7)	62 (14)
Bisphenol PH	ND	ND	101 (7)	63 (37)
**BPM** (Bisphenol M)	ND	73 (16)	115 (12)	77 (9)
**BPP** (Bisphenol P)	ND	56 (12)	90 (10)	61 (11)
4-Phenylphenol	ND	ND	102 (10)	75 (12)
Dichlorophen	24 (40)	121 (9)	137 (10)	88 (10)
Hexachlorophen	17 (91)	41 (74)	99 (10)	73 (26)
Bithionol	47 (59)	79 (41)	122 (7)	72 (18)
**TBBPA** (3,3',5,5',-Tetrabromobisphenol A)	57 (19)	102 (10)	119 (6)	78 (11)
**TBBPS** (Tetrabromobisphenol S)	50 (34)	72 (14)	87 (6)	61 (16)
Triclosan	95 (8)	122 (9)	132 (9)	94 (11)
2,4-Dichlorophenol	ND	ND	<5	93 (4)
2,4,6-Trichlorophenol	ND	ND	12 (26)	91 (10)
2,4-Dibromophenol	ND	18 (80)	25 (3)	83 (8)
2,4,6-Tribromophenol	ND	53 (24)	49 (4)	85 (9)
4,6-Dichlororesorcinol	117 (12)	126 (7)	114 (9)	85 (7)
Triclocarban	54 (20)	91 (6)	119 (6)	85 (5)
**Benzophenone-2** (2,2,4,4 Tetrayhydroxy-benzophenone)	40 (20)	93 (5)	103 (4)	87 (8)
2,4,4 Trihydroxy-benzophenone	56 (17)	90 (4)	101 (3)	85 (8)
**Benzophenone-6** (2,2'-Dihydroxy-4,4'-di-methoxybenzophenone)	77 (12)	80 (5)	87 (4)	87 (13)

**Dioxybenzone or Benzophenone-8** (2,2'-Dihydroxy-4-methoxybenzophenone)	33 (28)	86 (6)	91 (6)	93 (10)
**Benzophenone-1** (2,4-Dihydroxy-benzophenone)	96 (6)	107 (3)	105 (4)	92 (6)
4-Hydroxybenzophenone	112 (8)	110 (5)	106 (5)	89 (6)

ND = Not detected.

[Note 1] Due to weak signals/low recoveries, the matrix effect was not calculated

[Note 2] Overlapping precursor ion peaks were found in samples.

Table S10. Summary of EDC standard identification (n = 64) in three spiking levels of follicular fluid samples (1 ng, 5 ng and 25 ng/mL follicular fluid) through Compound Discoverer (CD) software.

	**Spiking level I (1 ng /mL follicular fluid)**	**Spiking level II (5 ng spike/mL follicular fluid)**	**Spiking level III (25 ng spike/mL follicular fluid)**
**Compounds extracted based on precursor ion** *(mass error ≤ 5 ppm and RT variation RT 0.1 min)*	40	56	62
**Compounds not extracted based on precursor ion** *(analyte peak below 3 times S/N or analyte lost during pretreatment)*	24	8	2
**Compounds correctly identified through Compound Discoverer** *(≥ 70% matching score and RT variation ± 0.1 min)*	26	45	53
**True positive identification rate** *(ability of CD to correctly identify all compounds present in the spiking samples)*	41%	70%	83%
**False negative rate** *(software not correctly identifying analytes within the spiking samples)*	59%	30%	17%

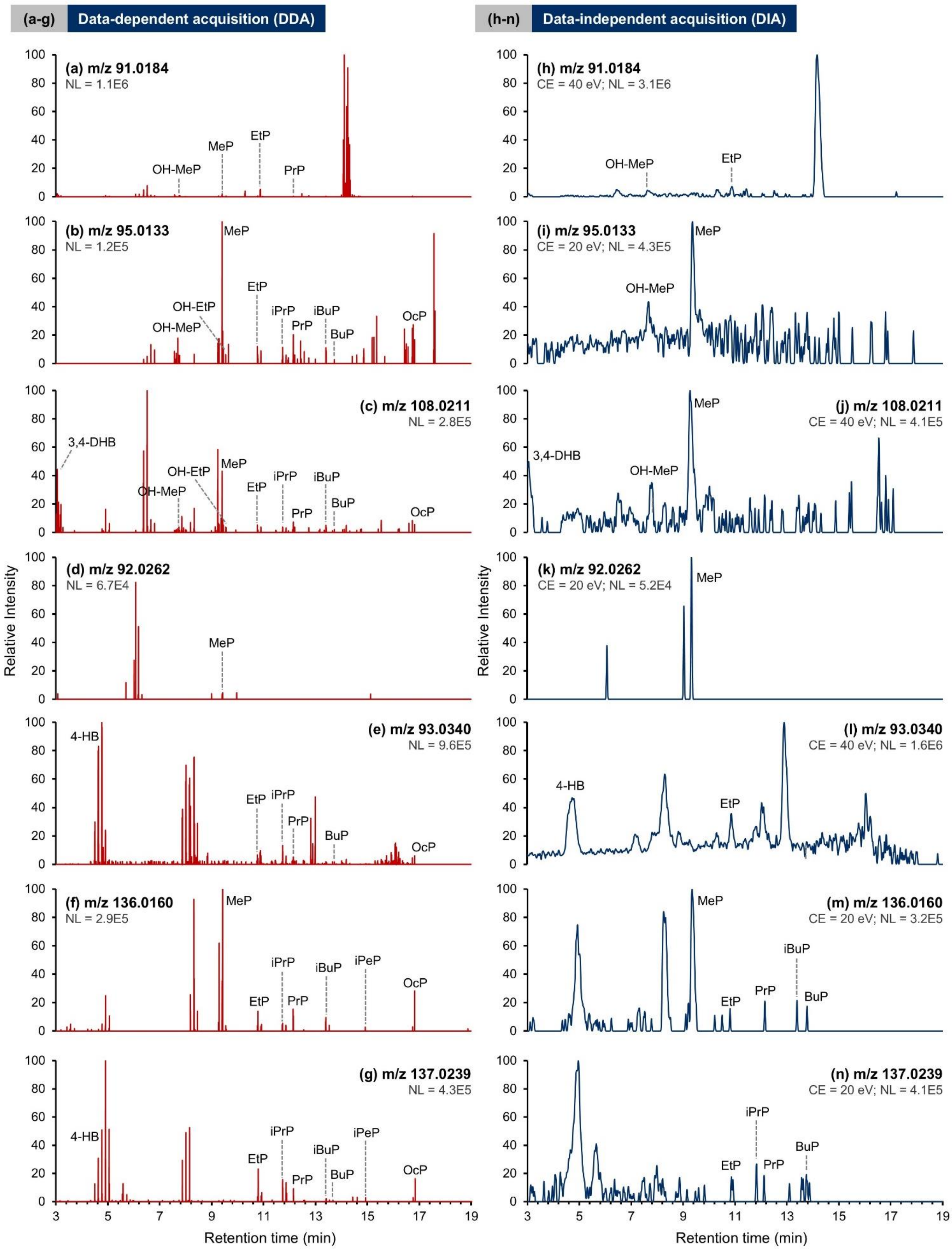

Figure S5. Extracted ion chromatograms (EICs) of paraben diagnostic fragmentation ions in **(a-g)** DDA and **(h-n)** DIA data of a pool follicular samples with a spiking concentration of 1 ng/mL. Mass error setting is 10 ppm.

5. Compound identification in non-spiked follicular fluid

Table S11. (2 pages) Parabens and phase I metabolites identified through diagnostic ions in hydrolyzed pooled follicular fluid. For some compounds listed as isomers (level 3 confidence), further structural proposal and retention time confirmations/predictions are shown in Table S13.

Identified Compound	Confidence level (CL)	Calculated Formula	Measured m/z	Mass error (ppm)	Measured RT (min)	Standard RT (min)	Observed diagnostic fragment ions
**MeP** (Methyl paraben)	1	$C_8H_8O_3$	151.0401	3.84	9.4	9.4	136.0165, 123.0088, 108.0217, 95.0139, 92.0269, 91.0190
**EtP** (Ethyl paraben)	1	$C_9H_{10}O_3$	165.0557	3.21	10.9	10.8	137.0244, 136.0165, 123.0089, 108.0217, 95.0139, 93.0346, 91.0190
**PrP** (Propyl paraben)	1	$C_{10}H_{12}O_3$	179.0714	3.24	12.2	12.2	137.0244, 136.0165, 123.0086, 108.0217, 95.0139, 93.0346, 91.0191
**OH-MeP** (Methyl 3,4-dihydroxybenzoate)	1	$C_8H_8O_4$	167.0350	3.41	7.7	7.7	152.0115, 139.0037, 124.0165, 123.0087, 111.0087, 108.0216, 107.0138, 95.0139, 91.0190
**OH-EtP** (Ethyl 3,4-dihydroxybenzoate)	1	$C_9H_{10}O_4$	181.0508	3.98	9.5	9.5	153.0193, 152.0115, 124.0166, 123.0088, 111.0087, 109.0294, 108.0216, 95.0139
**4-HB** (4-Hydroxybenzoic acid)	1	$C_7H_6O_3$	137.0244	3.87	4.7	4.7	93.0346
**3,4-DHB** (3,4-Dihydroxybenzoic acid)	1	$C_7H_6O_4$	153.0194	4.05	3.0	3.1	109.0294, 108.0217, 91.0189
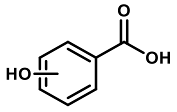 *Isomer of 4-HB*	3	$C_7H_6O_3$	137.0244	3.87	5.0	NA	93.0347
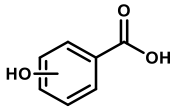 *Isomer of 4-HB [Note 1]*	3	$C_7H_6O_3$	137.0244	3.87	8.1	NA	93.0346
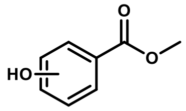 *Isomer of MeP*	3	$C_8H_8O_3$	151.0401	3.84	8.4	NA	136.0165, 108.0217, 95.0139, 93.0346
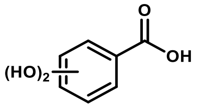 *Isomer of 3,4-DHB*	3	$C_7H_6O_4$	153.0194	4.05	6.2	NA	109.0294, 91.0190

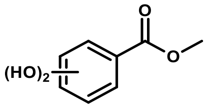 *Isomer of OH-MeP*	3	$C_8H_8O_4$	167.0350	3.41	6.5	NA	152.0115, 139.0035, 124.0165, 111.0089, 108.0217, 95.0140, 91.0190
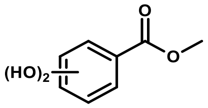 *Isomer of OH-MeP*	3	$C_8H_8O_4$	167.0350	3.41	8.5	NA	152.0115, 123.0085, 108.0217, 95.0140
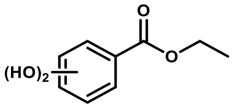 *Isomer of OH-EtP*	3	$C_9H_{10}O_4$	181.0507	3.42	8.4	NA	153.0193, 152.0115, 124.0165, 123.0089, 111.0087, 109.0295, 108.0217, 91.0190

[Note 1] This structure was later confirmed to be salicylic acid (2-HB) at level 1 confidence. See **Table S13** for details.

Table S12. Phase II metabolites of parabens identified through diagnostic ions in unhydrolyzed pooled follicular fluid.

Identified Compound	Confidence level (CL)	Calculated Formula	Measured m/z	Mass error (ppm)	Measured RT (min)	Modeled RT (min)	Observed diagnostic fragment ions at all CEs (10, 20 & 40 eV)
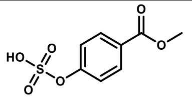 Methyl paraben sulfate	2	$C_8H_8O_6S$	230.9970	2.90	8.5	8.2	151.0400, 136.0165, 123.0091, 103.0217, 95.0139, 92.0269, 91.0191, 79.9571
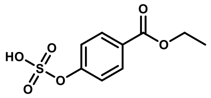 Ethyl paraben sulfate	2	$C_9H_{10}O_6S$	245.0126	2.53	10.4	10.1	165.0557, 136.0165, 137.0244, 123.0088, 108.0217, 96.9602, 95.0139, 93.0346, 91.0192, 79.9574
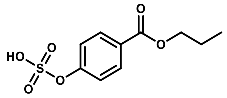 Propyl paraben sulfate	1 [Note 1]	$C_{10}H_{12}O_6S$	259.0283	2.59	12.2	11.7	179.0712, 137.0243, 136.0165, 123.0088, 108.0217, 96.9602, 95.0139, 93.0346, 91.0192, 79.9574
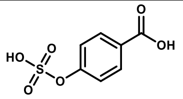 4-Hydroxybenzoic acid sulfate (or 4-Sulfooxybenzoic acid)	2	$C_7H_6O_6S$	216.9812	2.40	3.1	3.3	137.0243, 96.9602, 93.0346, 79.9573
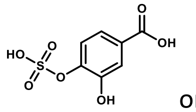 or 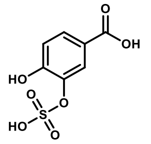 3,4-dihydroxybenzoic acid sulfate	3	$C_7H_6O_7S$	232.9756	0	2.6	1.1 and 1.3	188.9853 ($[M–CO_2]^-$), 153.0195, 109.0293, 108.0216, 96.9603, 79.9572
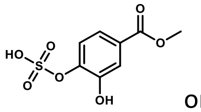 or 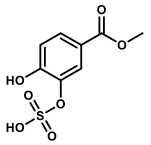 Methyl 3,4-dihydroxybenzoate sulfate	2	$C_8H_8O_7S$	246.9919	2.63	5.6	5.6 and 5.7	167.0349, 152.0014, 108.0125, 79.9571

[Note 1] Propyl paraben sulfate was assigned to level 1 confidence as its measured RT matched both the modeled prediction (11.7 min) and pure standard (12.2 min). Pure standards were not available for other identified sulfate conjugates.

Table S13. Selected phenols and metabolized organic acids in hydrolyzed follicular fluid identified through Compound Discoverer. Note that not all annotated compounds listed in this table are confirmed EDCs. Values listed under 'Standard RT (min)' indicate measured retention times of pure standards, and no model prediction was made for such compounds. For compounds without available standards [i.e., compounds with 'NA' under the column 'Standard RT (min)'], retention time model predictions were performed.

Annotated Compound	Confid. level (CL)	Calculated Formula	m/z	Measured RT (min)	Standard RT (min)	Model predicted RT (min)	Major fragments matching the library spectra
3,4-Dihydroxybenzoic acid	1	$C_7H_6O_4$	153.0194	3.0	3.1	NA	91.0190, 108.0217, 109.0294
4-Hydroxyhippuric acid	1	$C_9H_9NO_4$	194.0459	3.0	3.1	NA	65.0395, 74.0247, 93.0346, 100.0041, 130.9925, 150.0560
4-Hydroxybenzoic acid	1	$C_7H_6O_3$	137.0244	4.7	4.7	NA	93.0346
Hippuric acid	1	$C_9H_9NO_3$	178.0509	6.4	6.4	NA	132.0454, 134.0615
Methyl 3,4-dihydroxybenzoate	1	$C_8H_8O_4$	167.0350	7.7	7.7	NA	67.0189, 83.0140, 91.0191, 95.0140, 107.0141, 108.0219, 111.0087, 123.0088, 124.0165, 139.0037, 152.0114
Salicylic acid	1	$C_7H_6O_3$	137.0244	8.1	8.1	NA	65.0396, 93.0346
Monomethyl phthalate	1	$C_9H_8O_4$	179.0351	9.2	9.2	NA	107.0503, 121.0294, 135.0453
Methyl paraben	1	$C_8H_8O_3$	151.0401	9.4	9.4	NA	91.0190, 92.0269, 95.0139, 108.0217, 121.0296, 123.0088, 136.0165
Ethyl protocatechuate	1	$C_9H_{10}O_4$	181.0508	9.5	9.5	NA	59.0136, 91.0190, 95.0141, 107.0139, 108.0217, 109.0295, 111.0088, 123.0087, 124.0165, 152.0115, 153.0193
Monoethyl phthalate	1	$C_{10}H_{10}O_4$	193.0507	10.6	10.6	NA	107.0500, 121.0295, 121.0659, 134.0374, 147.0089, 149.0608
Ethyl paraben	1	$C_9H_{10}O_3$	165.0558	10.9	10.8	NA	65.0396, 91.0190, 93.0346, 95.0139, 108.0217, 121.0296, 123.0089, 136.0165, 137.0244
Propyl paraben	1	$C_{10}H_{12}O_3$	179.0714	12.2	12.2	NA	65.0396, 91.0191, 93.0346, 95.0139, 108.0217, 121.0293, 123.0086, 136.0165, 137.0244
Monoisobutyl phthalate	1	$C_{12}H_{14}O_4$	221.0821	13.1	13.0	NA	71.0502, 107.0502, 121.0294, 134.0373, 147.0088, 149.0971, 177.0920

Benzophenone-1	1	$C_{13}H_{10}O_3$	213.0558	14.4	14.3	NA	65.0031, 91.0190, 135.0088, 135.0193, 169.0658
3-Phenyllactic acid	2	$C_9H_{10}O_3$	165.0557	7.5 and 7.8	NA	7.9	72.9931, 91.0555, 101.0397, 103.0554, 117.0345, 119.0502, 147.0451, 178.0271
5-Methoxysalicylic acid	2	$C_8H_8O_4$	167.0351	8.5	NA	8.3	108.0217, 109.0249, 123.0451, 152.0116, 153.0149
Homovanillic acid	2	$C_9H_{10}O_4$	181.0507	9.9	NA	9.9	123.0452, 137.0608
4-Chlorophenol	2	C_6H_5ClO	126.9956	11.1	NA	10.4	91.0190
4-Chlorophenoxyacetic acid	2	$C_8H_7ClO_3$	185.0011	11.1	NA	10.8	91.0189, 111.0008, 126.9956, 141.0113
2-Hydroxycinnamic acid	2 and 3	$C_9H_8O_3$	163.0402	6.6 and 7.9	NA	8.5	65.0396, 91.0553, 93.0346, 117.0345, 119.0501, 135.0452
Paracetamol	3	$C_8H_9NO_2$	150.0560	3.6	NA	5.8	78.0349, 107.0376, 118.0299
2-Hydroxyhippuric acid	3	$C_9H_9NO_4$	194.0458, 194.0459	3.9 and 7.3	NA	5.3	65.0396, 93.0347, 108.0216, 121.0295, 137.0242, 150.0560
2-Hydroxyphenylacetic acid and 4-Hydroxyphenylacetic acid	3	$C_8H_8O_3$	151.0401	4.7	NA	7.5 and 7.9	77.0397, 93.0346, 95.0137, 95.0503, 107.0502, 121.0294, 123.0452, 123.0451
				5.4			77.0395, 79.0552, 93.0346, 95.0503, 106.0423, 107.0502, 121.0293
				6.5			79.0550, 106.0425, 107.0502
3,5-Dihydroxybenzoic acid	3	$C_7H_6O_4$	153.0194	6.2	NA	2.0	65.0396, 67.0189, 109.0295, 135.0089
2,4-Dihydroxybenzoic acid						5.1	
2,3-Dihydroxybenzoic acid						4.7	
2,5-Dihydroxybenzoic acid						3.9	
Vanillic acid	3	$C_8H_8O_4$	167.0350	6.5	NA	7.8	78.0348, 91.0190, 95.0139, 108.0217, 123.0451, 139.0043, 152.0115
4-Methylphenol	3	C_7H_8O	107.0503	6.6	NA	14.3	79.0553, 106.0424
Benzoic acid	3	$C_7H_6O_2$	121.0296	6.8	NA	8.2	93.0346, 108.0217
Ferulic acid	3	$C_{10}H_{10}O_4$	193.0506	8.9	NA	10.1	89.0395, 93.0347, 121.0295, 134.0373, 137.0244, 139.0401, 149.0611
4-Toluic acid	3	$C_8H_8O_2$	135.0452	9.1	NA	10.6	91.0553, 135.0451
4-Methylhippuric acid	3	$C_{10}H_{11}NO_3$	192.0667	10.3	NA	7.9	148.0768, 174.0562

Daidzein	3	$C_{15}H_{10}O_4$	253.0508	11.5	NA	15.2	91.0190, 132.0217, 133.0295, 135.0090, 153.0188, 169.0661, 180.0582, 195.0452, 208.0530, 223.0401
Genistein	3	$C_{15}H_{15}O_5$	269.0458	12.5	NA	15.2	63.0239, 91.0190, 95.0501, 107.0139, 133.0295, 135.0452, 159.0453, 180.0583

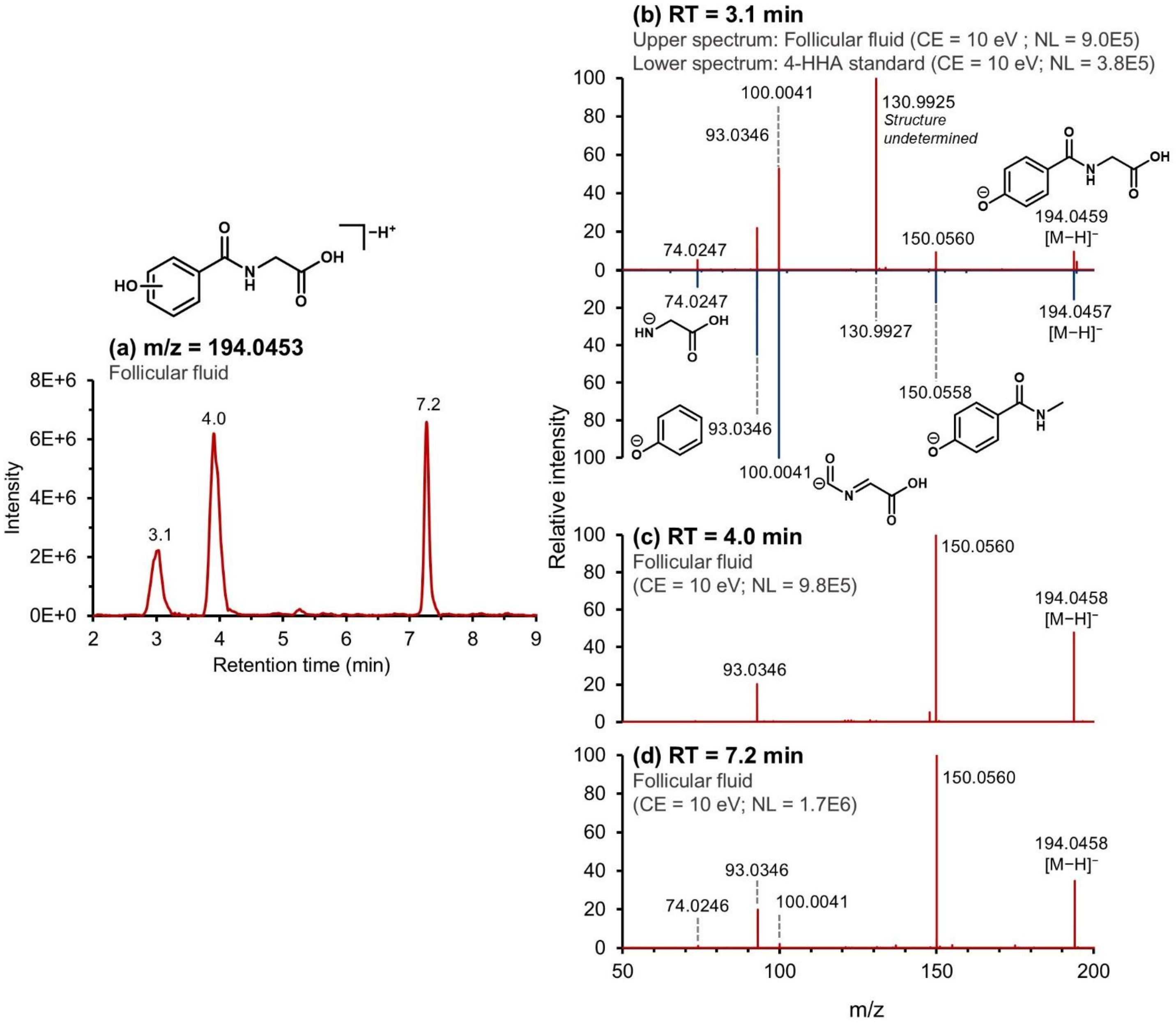

Figure S6. (a) Extracted ion chromatograms (EICs) of the precursor ion at m/z 194.0453 (mass error ± 5 ppm) detected in hydrolyzed follicular fluid, showing three peaks matching the MS and MS2 profiles of 4-hydroxyhippuric acid (4-HHA) in the mass spectral library. **(b-d)** MS2 spectra of the precursor ion (m/z 194.0453) detected in follicular fluid (red) eluting at 3.1, 4.0 and 7.2 minutes, respectively. Further MS2 fragmentation and retention time validation with a pure standard (lower blue spectrum in **b**) confirmed the peak at 3.1 min as 4-HHA with level 1 confidence.

References

(1) Meshref, S.; Li, Y.; Feng, Y.-L. Prediction of Liquid Chromatographic Retention Time Using Quantitative Structure-Retention Relationships to Assist Non-Targeted Identification of Unknown Metabolites of Phthalates in Human Urine with High-Resolution Mass Spectrometry. *J. Chromatogr. A* **2020**, *1634*, 461691.
(2) Feng, Y.-L.; Feng, J.; Baesu, A.; Martinez, V.; Li, Y. Development of a Quantitative Structure-Response Relationships to Estimate Concentrations of Plasticizer Metabolites in Urine without Reference Standards Using Non-Targeted Analysis with Liquid Chromatography High-Resolution Mass Spectrometry. *Anal. Chim. Acta* **2025**, *1364,* 344215.
(3) Feng, Y.-L.; Liao, X.; Grenier, G.; Nguyen, N.; Chan, P. Determination of 18 Phthalate Metabolites in Human Urine Using a Liquid Chromatography-Tandem Mass Spectrometer Equipped with a Core–Shell Column for Rapid Separation. *Anal. Methods* **2015**, *7*, 8048–8059.
(4) Bienvenu, J.-F.; Provencher, G.; Bélanger, P.; Bérubé, R.; Dumas, P.; Gagné, S.; Gaudreau, É.; Fleury, N. Standardized Procedure for the Simultaneous Determination of the Matrix Effect, Recovery, Process Efficiency, and Internal Standard Association. *Anal. Chem.* **2017**, *89*, 7560–7568.
(5) Łozowicka, B.; Rutkowska, E.; Jankowska, M. Influence of QuEChERS Modifications on Recovery and Matrix Effect during the Multi-Residue Pesticide Analysis in Soil by GC/MS/MS and GC/ECD/NPD. *Environ. Sci. Pollut. Res.* **2017**, *24*, 7124–7138.
(6) Rajski, Ł.; Petromelidou, S.; Díaz-Galiano, F. J.; Ferrer, C.; Fernández-Alba, A. R. Improving the Simultaneous Target and Non-Target Analysis LC-Amenable Pesticide Residues Using High Speed Orbitrap Mass Spectrometry with Combined Multiple Acquisition Modes. *Talanta* **2021**, *228*, 122241.
